# Sleep medicine: Practice, challenges and new frontiers

**DOI:** 10.3389/fneur.2022.966659

**Published:** 2022-10-14

**Authors:** Liborio Parrino, Peter Halasz, Anna Szucs, Robert J. Thomas, Nicoletta Azzi, Francesco Rausa, Silvia Pizzarotti, Alessandro Zilioli, Francesco Misirocchi, Carlotta Mutti

**Affiliations:** ^1^Department of General and Specialized Medicine, Sleep Disorders Center, University Hospital of Parma, Parma, Italy; ^2^Szentagothai János School of Ph.D Studies, Clinical Neurosciences, Semmelweis University, Budapest, Hungary; ^3^Department of Behavioral Sciences, National Institute of Clinical Neurosciences, Semmelweis University, Budapest, Hungary; ^4^Division of Pulmonary, Critical Care and Sleep, Department of Medicine, Beth Israel Deaconess Medical Center and Harvard Medical School, Boston, MA, United States; ^5^Department of Medicine and Surgery, Unit of Neurology, University of Parma, Parma, Italy

**Keywords:** sleep, sleep disorders, sleep medicine, sleep diseases, sleep medicine evolution

## Abstract

Sleep medicine is an ambitious cross-disciplinary challenge, requiring the mutual integration between complementary specialists in order to build a solid framework. Although knowledge in the sleep field is growing impressively thanks to technical and brain imaging support and through detailed clinic-epidemiologic observations, several topics are still dominated by outdated paradigms. In this review we explore the main novelties and gaps in the field of sleep medicine, assess the commonest sleep disturbances, provide advices for routine clinical practice and offer alternative insights and perspectives on the future of sleep research.

The evolution of sleep medicine is simultaneously uneven, turbulent, exuberant, exciting, disappointing, confusing, and exhilarating. There is a continuous push and pull between the several forces, which typically sculpt a field, especially a relatively new one. For instance, the hurry of science leads the evolution of formal treatment guidelines. This is amplified by the fact that the boundaries of sleep medicine and science are nearly infinite, with inputs from a range of specialties and the confluence of views of individuals and entities with very divergent visions. This article attempts to capture the excitement of clinical and translational sleep medicine, explore the existing gaps and needs, integrate cutting edge technologies, and propose some solutions.

## Insomnia and the disturbed, unhappy sleeping brain

In 2005, the inaugural issue of the Journal of Clinical Sleep Medicine reviewed the history of sleep research in the United States, where Sleep Medicine had recently been recognized as a specialty of medicine (1). The article chronicled the development of sleep science and sleep organizations over the previous 70 years, highlighting the progressive increase of knowledge concerning the physiology of sleep, circadian biology and the pathophysiology of sleep disorders. In the closing session, entitled The Future of Sleep Medicine, the authors pointed out the major challenges linked to the technological advances of our society: *The development of artificial lighting has contributed greatly to the problem of sleep deprivation. We are now able to run factories, stores and the Internet 24/7; thereby, increasing the efficiency of economic activity. However, we have not been able to adapt human circadian rhythms and need for sleep to meet either the economic demands of society or the socially desired preferences of individuals that result in insufficient sleep. “On demand” sleep and/or alertness will likely become a major goal and challenge for the field of Sleep Medicine. Pharmacological treatments to promote sleep predisposition or, conversely, to stimulate wakefulness, are available but have limited efficacy. Adjusting the timing of light/dark exposure effectively shifts circadian rhythms but it is slow, difficult to regulate and relies on patients' compliance. New methods, technologies and treatments must be developed to meet the demands for alertness and sleep*.

Fifteen years after the acceptance of sleep medicine as a distinct speciality, the interplay between sleep and society remains an open and unsolved dilemma. In particular, insomnia remains a common disorder in the general population, with variable prevalence-estimates in the different age groups and in relation to socio-demographic, clinical, and psychosocial parameters. A systematic review published in 2019 (2) established that: (a) approximately one third of adults (>18 years of age) reported dissatisfaction with their sleep and at least one symptom of insomnia. It has also been shown, that: (a) the use of prescription sleep aids, particularly non-benzodiazepines and (off-label) antidepressants, has risen significantly over the last 20 years; (b) 70% of patients using a prescription sleep aid continue to do so at 1-year follow-up despite lack of significant improvements in sleep compared to non-users; (c) up to 60% of sleep aids used by adults for insomnia, are over-the counter products (3–5). According to the European guideline for the diagnosis and treatment of insomnia (6), there is very little information in terms of the management and persistence of chronic insomnia. Moreover, the prevalence of insomnia and of hypnotic usage (benzodiazepines and benzodiazepine receptor agonists) varies largely from one European country to the other. Several national surveys in general practice (GP) or medical specialty settings have been conducted. In Italy (7), insomnia was reported by 64% of all interviewed patients; more than 50% of GP patients had insomnia in Norway (8) and in Germany (9) where the prevalence of having taken a hypnotic drug at least once, increased from 4.7 to 9.2% from 2009 to 2016 (10). A recent analysis on the assessment and management of insomnia illustrates numerous gaps in research carried out to date (11): (a) inadequate information on the specific effects of various components of Cognitive Behavioral Therapy (CBT-I) which might allow greater treatment efficiency and tailoring; (b) lack of double-blind, placebo-controlled, randomized trials demonstrating the efficacy of pharmacological treatments in children or adolescents with insomnia; (c) lack of rigorous investigations on a number of agents commonly used to treat insomnia in clinical practice, including trazodone, quetiapine and gabapentin; (d) lack of pharmacological treatment of insomnia in the setting of fragile medical conditions such as dementia, mild cognitive impairment and substance use disorders; (e) necessity to move to greater personalization in clinical practice and therapy. These considerations, combined with the extension of the daytime due to electric lighting exposure, internet connection, digital revolution, shift work, geographical and social jet-lag; suggest that, at least in the field of insomnia, the future of sleep medicine is still unclear. With the arrival of SARS-Cov 2, the picture has become even more complicated.

## Insomnia in the time of COVID

The COVID-19 pandemia has enhanced the difficulty to find a set-point between the laws of physiology and the rules of modern life. The first lockdown triggered worldwide a sharp increase in sleeping problems. In a survey conducted in Italy and Belgium (12), sleep timing was significantly delayed, time spent in bed increased, and sleep quality was markedly impaired. The most vulnerable individuals appeared to be women, subjects experiencing more negative mood and those perceiving the pandemic situation as highly stressful. Sleep quality and timing underwent significant modifications especially in unemployed participants. In contrast, positive mood showed a protective effect against the risk of experiencing poor sleep quality. In a French general public sample, COVID-19-related worries and loneliness were major contributing factors to clinical insomnia (13). During the shutdown in China, a survey revealed that insomnia was highly prevalent and associated with COVID-19 outbreak–related psychological reactions and poor sleep hygiene (14). These findings indicate that both excessive social mobility (pre-COVID) and prolonged social isolation (pandemic lockdown) can jeopardize sleep quality, introducing additional caveats in the competition between Nature and Culture. According to Immanuel Kant: There is nothing more unsociable than Man, and nothing more sociable: unsociable by his vice, sociable by his nature. A relevant association between insomnia severity and confinement, loneliness, perceived stress, anxiety and/or depressive symptoms have been confirmed by numerous studies worldwide, highlighting the need for large-scale social intervetion against dramatic socio-health crises.

## The semantic shades of insomnia

In the Latin dictionary, the definition of insomnia is not confined to the difficulty of starting and/or maintaining sleep, but also includes the inability to sleep well due to nightmares or agonizing dreams. Dream is said somnium (neuter), but there is also the word insomnia (feminine) to mean sleeplessness. In the Aeneid, Dido reveals to her sister Anna: these dreams stir and frighten me (insomnia terrent). Virgil uses insomnium as a plural noun—insomnia—to signify frightening (terrent) dreams. Accordingly, individuals frequently experiencing nightmares, report compromised sleep quality, poor daytime mood and functioning. The last 5 min of REM (rapid eye movements) sleep before awakening were analyzed individuals experiencing nightmares, and a comparison of REM sleep was made subjects with non-nightmare dream episodes and non-dreaming control individuals (15). Overall, there was no general difference in autonomic activation of nightmare sufferers compared to control individuals. However, when nightmare persones experienced nightmares, the autonomic activation was markedly increased compared to their own non-nightmare dreams and, to some extent, to control's dreams demonstrating an increased autonomic activation associated to nightmares. These findings support the role of vegetative responses in impaired self-reported sleep quality.

## Insomnia phenotypes: The sleep train

Insomnia definition has dynamically evolved throughout the years. Insomnia can be either acute or chronic; organic or non-organic; initial, middle and late-night in terms of occurrence; paradoxycal or psychophysiological, associated with misperception or objectively confirmed, isolated or associated with coexistent sleep disorders. Insomnia may affect patients suffering from various neurodegenerative disorders, likely reflecting differential pathogenetic mechanisms and frequently impacting on patients's caregivers as well. Sleep fragmentation is also commonly observed in the acute/subacute phase of neurological disorders such as stroke, traumatic brain injury, encephalitis.

**Figure 1:**
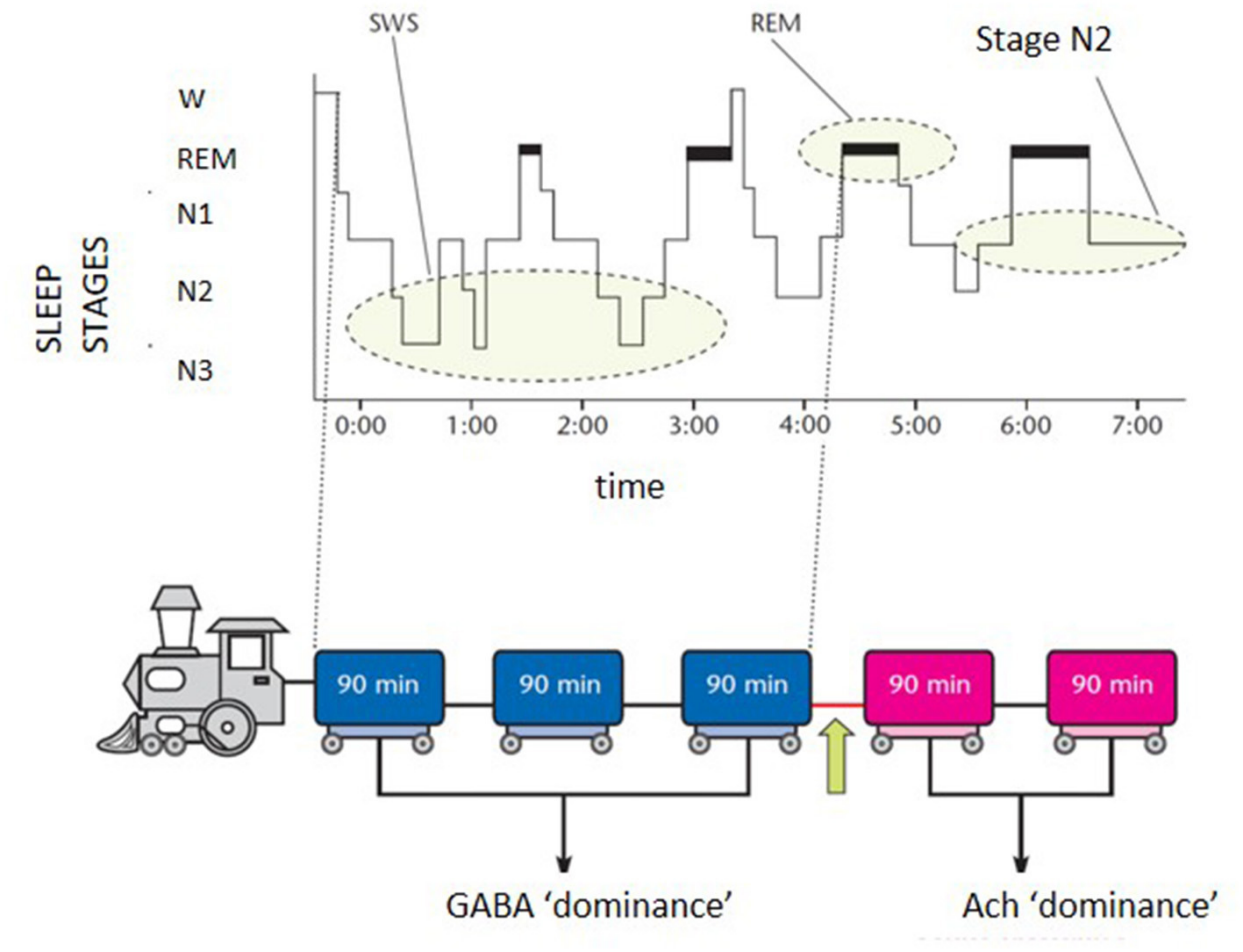
Schematic representation of the “sleep train”. GABA, gamma-aminobutyrate; Ach, acetylcholine.

The scenario is far from homogeneous and the subgrouping of insomnia disorder is an ongoing challenge in sleep research. Research investigation should harmonize definitions and categories of the heterogeneous field of insomnia disorder. In the effort of “phenotyping” the insomnia disorder, a practical schematic representation for both clinicians and patients can be portrayed by the “sleep train”. With the sleep histogram, the succession of stages and cycles can be imagined as a train composed of 5 carriages (Figure 1). Each wagon, represented by the complete sleep cycle, lasts about an hour and a half. The first three carriages, which constitute the so-called “core sleep”, are mainly controlled by the gamma-aminobutyric acid (GABA), a sedative neurotransmitter. The last two wagons compose the traditionally defined “optional or complementary sleep” modulated by activating neurotransmitters including acetylcholine, which prepares the brain to morning awakening. The transition point between the two types of sleep, placed between the third and fourth carriage, coincides with a delicate phase of sleep continuity and often represents the middle-of-the-night awakening for many insomniacs. During the first 3–4 h of the night the GABA carriages recharge the brain battery attenuating the tendency to doze-off during the day. Lack of excessive daytime sleepiness (EDS) is often found in insomniacs who sleep soundly for at least 4 h in the first part of the night. However, detachment of the hook between the 3rd and 4th wagon can derail the last two cars of sleep, which travel under the dominion of acetylcholine, fundamental for memory consolidation and muscle activity. Therefore, when a patient complains of sleep maintenance insomnia, it becomes crucial to clarify the time at which nocturnal awakenings occur. If these prevail in the early part of the night, the diurnal repercussions (excessive sleepiness) will be different from what happens when only the last 2–3 h of sleep are compromised (fatigue, difficulties in attention and concentration, poor or absent dream activity). Therapeutic strategies will differ according to the sleep wagons that need stronger protection. For example, GABA-ergic drugs, which sustain sleep continuity when the initial wagons are frail, become unsuitable for late sleep disruption and their administration under the acetylcholine dominance (wagons 4 and 5) represents a neurochemical inconsistency.

## Insomnia and sleep-disordered breathing

Sleep and breathing are rarely at peace with each other, disordered breathing being a common stressor of the sleep system. While the official guidelines promote the idea that apnea and insomnia are separate, there is mounting evidence that the two can coexist and interact in complex ways. One term surfacing increasingly is “COMISA”, Comorbid Insomnia and Sleep Apnea. This is especially true for central/high loop gain/NREM-dominant sleep apnea, with very prominent unstable NREM (non rapid eye movements) sleep. Low arousal threshold and arousals in general tend to destabilize NREM sleep, contributing to sleep-breathing instability and worsening of sleep apnea. The time is here to carefully and consistently estimate the state of sleep-breathing in the evaluation and management of chronic insomnia.

## Insomnia as a chamaleontic condition

Although in many cases chronic insomnia represents the main sleep disorder, there are many other cases where insomnia is only the tip of the iceberg, being the most visible consequence of a more complex sleep disturbance.

Basically, any sleep disorders can lead to clinically significant insomnia: from periodic limb movement disorder to NREM sleep parasomnias or sleep related epilepsies.

A detailed sleep anamnesis, enriched with instrumental evaluation (e.g., cardio-respiratory recording, video-polysomnography, actigraphy.) is essential to avoid misinterpretation.

According to the European guidelines the diagnostic procedure for insomnia should always include a sleep history inclusive of sleep habits, sleep environment, work schedules, circadian factors, the use of validated sleep questionnaires and/or sleep diaries, evaluation of somatic and mental health and a physical examination (6).

## Insomnia and CBT-I

Insomnia has frequent medical and mental health comorbididities, including post-traumatic stress disorder, depression and psychosis. Cognitive behavioral therapy for insomnia (CBT-I) is a safe and effective treatment for chronic insomnia in the context of various comorbid conditions. CBT-I is considered the first-line treatment for chronic insomnia in adults. A pharmacological intervention should be considered when CBT-I is not sufficiently effective or not available, mainly based on either short-acting benzodiazepines, benzodiazepine receptor agonists and/or antidepressants. Short term protocols with medications are recommended (ideally ≤ 4 weeks). It is effective in improving sleep and functioning in military settings including active duty-service members to older veterans with complex presentations including post-traumatic stress disorder, depression, sleep apnea, and chronic pain. Data evaluating CBT-I frequently focus on women service-members or individuals with substance abuse. While meta-analyses demonstrate the value of CBT-I, they also note significant heterogeneity. The variability in CBT-I components across trials makes it difficult to determine which aspects are the most responsible for the observed benefits. Future research is needed to better establish the effectiveness of CBT-I in patients with comorbid conditions, as well as on treatment sequencing and alternative methods of CBT-I delivery. In the treatment of insomnia, CBT-I and hypnotic drugs carry advantages and limitations of their own. Medications with specific indications for insomnia produce rapid symptomatic relief, but there is little to no evidence that sleep improvements are maintained after drug discontinuation or during long term use. Conversely, CBT takes longer than drugs to produce sleep improvements, but benefits are well-sustained over time. In the acute phase of CBT-I, adding pharmacotherapy may have a slightly better effect compared with CBT-I alone, if the medication is discontinued in the maintenance phase of CBT-I. Moreover, pharmacotherapy is not indicated for chronic use.

Unfortunately, CBT-I is not available in several countries, even though it carries multifold contradictions.

Several sleep disturbances are caused by wrong habits (busy lifestyles, sedentarism and weight gain, malnutrition and junk food, smoking, alcohol and drug abuse, shift-work, social jet-lag), and the so-called “organic” sleep disturbances, i.e., nocturnal breathing disorders, can co-occur with insomnia related to post-traumatic stress disorder, chronic painful conditions or even cancer. All such combinations are in favor of CBT-I treatment, which aims at reframing misconceptions and dysfunctional thoughts. In many cases, patients with primary insomnia experience subjective improvement with CBT-I: less time to fall asleep, more time spent asleep and waking up less during sleep. Treatment generally takes 6–8 lessons and may be as short as two sessions when given by a primary care doctor. In addition, online resources and smartphone applications offering digital CBT-I are available. Despite these promising opportunities, the question remains: is the therapeutical power of CBT-I really effective against labor constraints, global mobility, internet connection, round-the-clock consumption and production, daily menances against life rhythms and enhancement of waking hours (16). In other words, how effectively can CBT-I stand the challenge of a non-stop sleep-shrinking society? The uneven conflict between dwarfs (CBT-I) and giants (cultural forces) sends a sign of weakness to drug companies seeking for new neurochemical approaches to treat sleeplessness.

If insomnia symptoms can be effectively solved by easy-to-apply and short-lasting techniques of relaxation combined with educational and cognitive interventions, then why huge investments should be dedicated to discover new agents for a sleep disorder which can be managed by a professional psychological approach? Mutatis mutandis, would the managers and stakeholders of pharmaceutical industry dedicate a substantial research and development budget for a new medication against cancer if the latter could be treated by psychological interventions? These questions do not dampen or mortify the positive role of CBT-I, but query the field of sleep experts and international societies who continue to publish guidelines establishing that objective measures, i.e., polysomnography, neuroimaging, biomarkers are useless or unnecessary for the diagnosis and management of insomnia.

Finally, CBT-I present some disadvantages: its efficacy strongly depends on patients' cooperation, it does not work for all types of chronic insomnia disorder, it is time consuming, it is not easily available everywhere and is an expensive approach. Finally, as for any kind of counseling, it strongly depends on therapists' ability.

## New wine in old wineskins

Compounds that people take to treat insomnia include dietary supplements and over-the-counter sleep aids (for which prescription is not required), off-label sedating medications and approved medications for insomnia. Agents used for insomnia promote sleep by means of different mechanisms: enhancing GABAergic neurotransmission, antagonizing receptors for the wake-promoting monoamines, or binding the melatonin receptors (17). Orexin receptor antagonists comprise a new class of hypnotic drugs, which promote sleep by decreasing orexin-associated CNS arousal (18). The mechanism of action targets a region of the hypothalalmus involved in the regulation and sleep and wakefulness. An important decrease or total loss of orexin-containing neurons has been reported in brains of patients with narcolepsy. Among orexin-receptor antagonists, Suvorexant is available on the US market. Compared to placebo, 1 month of treatment with Suvorexant improves sleep to a greater extent as assessed by the insomnia severity index (ISI) with dose-dependent effects. In particular, remission is reported in 30% of patients using 20/15 mg (non-elderly/elderly) and in 35.5% of patients using 40/30 mg (non-elderly/elderly) of the active compound. The ratio of improving patiens increase to 48 and 56%, respectively when medication is used for 3 months. Although better than placebo, these findings indicate that Suvorexant warrants a clearcut improvement only in 30–56% of patients with insomnia. Furthermore, there is considerable variability among individuals and there may be a delay following a high-fat meal. Finally, the elimination half-life is ~12 h. After decades of recommendations promoting the use of hypnotic agents with short half-life in order to avoid residual hangover effects, the new wine of pharmacotherapy offers an old wineskin perspective. Hopefully, new anti-orexinergic compounds (daridorexant) with shorter half-lives and limited side-effects will be soon available to explore alternative pathophysiological and neurochemical pathways in the treatment of insomnia.

Another potential approach for chronic insomnia is the use. Data on pediatric patients are still fragmentary, adult insomniac patients seem to benefit from short-term protocols with nabilone and dronabinol, although it seems that long-term therapy might impair sleep quality (19).

## Poor sleepers: Orphaned patients

There is a category of orphaned patients who do not belong to a specific category of sleep disorders. They are people who generally complain of sleeping badly and feeling tired and poorly rested during the day. However, if they carry out a PSG recording, the examination shows no significant alterations, at least for the official standards. They are generally adults and their conventional objective measures (sleep efficiency, sleep stages) remain within the age-related ranges. Sometimes, an increased amount of nocturnal awakenings are recorded but we're not talking about classical imsomniacs with difficulty initiating or maintaing sleep. These subjects are all-night sleepers but they wake up poorly refreshed and with the sensation of a restless night. Still, the apena-hypopnea index is < 5/h and the periodic limb movement index remains < 15/h. Both the 3rd International Classification of Sleep Disorders and DSM-V include “non-restorative sleep” in the category of insomnia, but so far an approved marker of sleep quality is lacking. Unfortunately, in several countries, if you are not affected by sleep apnea or PLM you are addressed to a psychologist/psychiatrist and not to a sleep specialist. In some cases, a CBT-I, when available, can improve the symptoms, but once again we are neglecting the core message of the issue: what are the organic bases of poor sleep? In a study conducted on 385,292 British men and women, “sleep scores” were assigned on a scale of zero to five giving one point for having each of five indicators of healthy sleep: being an early bird, sleeping 7–8 h a night, having no insomnia, not snoring and not being sleepy during the day. The scores depended on self-reports of sleep behavior. Over the following 8 years, researchers found that the lower the sleep score, the higher the person's risk for coronary heart disease or stroke. Compared to the poorest sleepers, those who scored 5 had a 34% reduced risk for both coronary heart disease and stroke (20). If subjective poor sleep may increase the risk for cardiovascular disease, then there must be underlying mechanisms promoting a shift toward autonomic activation. A reliable candidate is the arousal system during sleep. Sudden drops in pulse wave amplitude (PWA) measured by pulse oximetry during sleep are commonly associated with simultaneous arousals. When a total of 1,085 PWA drops (below 20%) from 10 consecutive sleep recordings were analyzed, a significant increase in EEG power density in all frequency bands was found during PWA drops (*P* < 0.001) compared to before and after drop. Even in the absence of conventional EEG arousals, drops in PWA were associated with a significant increase in EEG power density, suggesting that these events can be used as surrogate markers for changes in cortical activity during sleep (21). As a confirmation, analyzing the PSG recordings of 20 male individuals with obstructive sleep apnea (OSA) the pulse wave amplitude drops (below 30%) during respiratory events were quantified and the combined or separate occurrence with the A phases of CAP (cyclic alternating pattern) was measured (22). A dual response (A-phase associated with a pulse wave amplitude drop) was the most frequent finding (71.8% in total sleep time) for all types of respiratory events, with a progressive reduction from apneas to hypopneas and flow limitation events. The highly significant correlation between CAP A-phases and relevant pulse wave amplitude drops (*p* < 0.0001) suggests a possible role of autonomic arousals as a marker of cerebral response to respiratory events and confirms the significance of PWA drops as indirect electrophysiological biomarkers of sleep instability. Thus, it would be interesting to explore the advantage of PWA (and CAP) in the classification of insomnia phenotypes.

**Figure 2:**
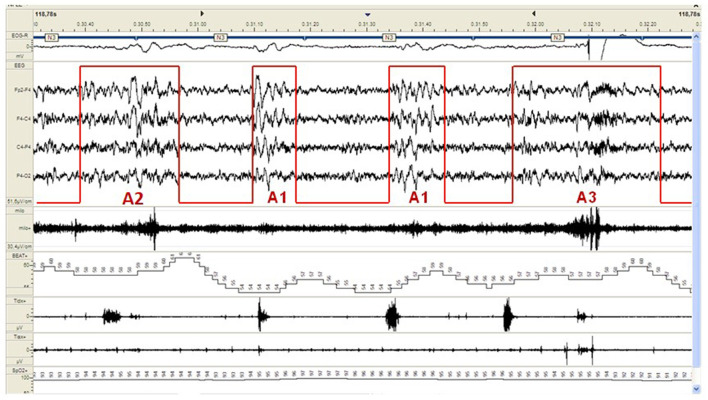
Example of a CAP sequence (CAP phases A highlighted in red) during stage N3 of NREM sleep in a patient affected by periodic limb movement disorder (PLMD). Note that the more disturbing leg movements (right part of the figure) are associated with CAP subtype A3. CAP subtypes A1 have a milder cardiovascular impact.

CAP represents a pivotal component of the dynamic sleep structure and can be considered a polysomnographic hallmark of sleep instability. CAP is organized in cycles, with subsequent cycles composing a CAP sequence. Depending on the presence or absence of CAP cycles, sleep itself can be divided into “CAP sleep” and “non-CAP sleep”. Each CAP cycle can be subdivided in a phase A (activation phase) and a following phase B (de-activation phase). Phase A can be further classified in three subtypes: A1, A2 and A3, according to reciprocal proportion of slow waves and fast rhythms (see Figure 2). The distinction is not trivial as CAP subtypes A1 typically boost SWS and reinforce sleep propensity, whilst CAP subtypes A2 and A3 usually translate a tendency toward REM sleep and/or prepare the brain for an awakening. The utilization of CAP metrics ensures a deeper understanding of sleep kinetic troughtout the night, however, as CAP analysis is highly demanding and time-consuming, not all the sleep laboratories can guarantee its measurement in everyday clinical practice.

Novel methods to measure the level of NREM sleep instability, including cardiopulmonary coupling (CPC) spectrogram, have been developed and proved useful in tracking the effect of treatment (23). According to a recently published review of the available portable systems (2021), the EEG-based systems are the most accurate, while the photoplethysmography-based systems are simpler and better suited for wearable monitoring. Perhaps, time is ripe to entrust poor sleepers to a clinical family including CAP metrics and vegetative monitoring in the conventional sleep scoring procedures.

Literature is still lacking of clear “endoptype models” for chronic insomnia disorder, as pathophysiological mechanisms related to this condition are currently largely unknown and, so far, diagnosis is based on clinical evaluation. In this perspective, we suggest that the utilization of CAP metrics might help to reveal endotypes and subendotypes of this condition, while, concurrently, it might help to categorize various insomnia phenotypes.

## Restless leg syndrome and periodic limb movement disorder

Sleep, at any age, can be perturbed by numerous sleep-related movement disorders: a group of conditions characterized by simple, sometimes stereotyped, movements that can hamper sleep continuity and quality. The category of sleep-related movement disorders of the International Classification of Sleep Disorders (24) includes restless leg syndrome (RLS), periodic limb movement disorder (PLMD), propriospinal myoclonus at sleep onset, sleep-related leg cramps, bruxism, sleep-related rhythmic movement disorder, benign sleep myoclonus of infancy and the so-called “isolated symptoms and normal variants” comprehensive of excessive fragmentary myoclonus, hypnagogic foot tremor, alternating leg muscle activation and sleep starts. More recently a novel independent pediatric sleep-related movement disorder named restless sleep disorder (RSD) has been categorized (25).

## PLMD and RLS diagnostic criteria

RLS and PLMD are the commonest, frequently overlapping, sleep-related movement disorders in adulthood, with a prevalence estimated 5–15% and 4–11%, respectively (26, 27).

PLMs are repetitive, stereotyped, non-epileptiform, involuntary movements during sleep, usually involving the lower limbs and ending with a spinal cord flexor reflex-like motor pattern (28). The movements last 0.5–10 s, repeating at 5–90 s intervals. At least 4 consecutive movements define a PLM sequence (24). The PLM index is the total number of PLMs/total sleep time, and the cut-off values are fixed at >5/h in children and >15/h in adults. The definition of PLMD requires the instrumental motor findings associated with clinical consequences (insomnia and/or excessive daytime sleepiness) (29). In 2016, a task force of the Word Association of Sleep Medicine (WASM) revised the PLMD criteria to prevent “over-diagnosis” and promote the development of reliable automatic scoring (30). According to the WASM criteria either shorter (< 10 s) or longer (>90 s) intermovement intervals interrupt a series of PLMs (“periodicity criteria”), while the morphology of Leg Movements (LMs) needs to be evaluated to discard irrelevant or irregular movements; and in conditions involving predominantly one side e.g., hemiparesis, a separate unilateral analysis is recommended (30). The current rules for PLMs scoring suggest to exclude those movements from LM count linked (within 0.5 s) to a phasic respiratory event (29). Application of these criteria markedly reduces the frequency of PLMD diagnosis, confirming the risk for overestimation of the condition applying traditional scoring rules (31). An alternate view is that ALL movements should be accurately identified and tabulated to estimate the burden of motor activation. Clearly there are “kickers and non-kickers” for any given degree of sleep apnea, and we should better study these associations and the impact of concomitant neural subsystem activation, on physiological and clinical outcomes.

RLS diagnosis is based on four core clinical criteria (instrumental confirmation is not required): (1) an urge to move limbs with a typical unpleasant sensation; (2) symptoms worsening with inactivity; and (3) partially or totally relieved by movement; (4) worsening in the evening/night (32). Common mimics include restlessness of other sorts (psychiatric disorders, drugs) and sensory discomfort due to peripheral neuropathy, myelopathy or vascular diseases (33).

## Circadian/ultradian distribution of PLM and RLS

PLMs are largely prevalent during stage N1 and N2 of NREM sleep (34), progressively declining in deeper sleep and, even more, in REM sleep. The majority of LMs occur during the first sleep cycles and progressively declines overnight. This overnight/ultradian pattern might correlate with the circadian rhythm of core body temperature, melatonin dynamics, hormonal changes and dopamine activity (34–36). Similar circadian variations in RLS severity have been observed, significantly correlating with subjective vigilance level, core body temperature and salivary melatonin (37). Based on these findings, an inhibitory effect of melatonin toward dopamine secretion has been hypothezed. Intringuingly, the predictable circadian distribution of PLMs progressively disappears with aging; after the age of 75 years, the involuntary movements typically persist throughout the entire night, losing their ultradian pattern (38).

## Age-dependent dynamics of PLM and RLS

Recent data demonstrate a para-physiological age-related increase of PLM index across lifespan: its value starts to grow after age 10 years (mean value 4.5 ± 4.46/h), rises up to 25.7 ± 10.9/h at age 40 years, reaches 35.6 ± 23.3/h at 60 with a final increase over the age of 80 years (39).

However, when additional features are taken into account - such as the Periodicity Index (PI) - lower values of PLM index emerge. This point suggests a distinct (maybe not pathological) significance of these “non-periodic” movements during sleep, that are probably influenced by numerous external of internal factors perturbing sleep (e.g., coexistent sleep disturbances, changes in sleep stages that take place with aging), while the more “periodic” movements might reflect, in the youngest, the maturation of brain connections in parallel with daytime experiences (40, 41). Hence, to ensure a reliable clinical evaluation, PLM index should always be related to patient's age and additional features such as PI may ease the recognition of the more disturbing LMs.

The lifetime course of PLM index (PLMI) and PI does not overlap, as PLMI progressively increases with aging; PI reaches a plateau at around 15–25 years of age and remains somewhat stable up to 65 years. These “ontogenetic” differences suggest that the two phenomena mirror distinct neurobiological mechanisms (38).

Conversely, there is an impressive overlap between the typical PLMI curve and the nocturnal distribution of sleep instability, which can be measured using the CAP metrics (42). PLM sequences trigger powerful arousal intrusions during sleep, represented by CAP subtypes A2 and A3, and, at the same time, CAP cycles associated with PLMs last usually longer (42), suggesting a reciprocal influence between CAP dynamics and PLM.

Interestingly, in some cases, pharmacological interventions can markedly reduce LMs with no effect on sleep instability (e.g., dopaminergic drugs) and, vice-versa, some other drugs (e.g., clonazepam) can ameliorate CAP rate with no variations of PLMI (43).

RLS can either coexist with PLM or occur independently, especially in older people. RLS may affect children, adults and the elderly. Various lifetime changes (including pregnancy and breastfeeding) may enhance or provoke RLS symptoms' exacerbation. Both early-onset (frequently related to genetic predisposition) or late-onset variants exist (44). RLS diagnosis in the elderly may be particularly challenging due to the coexistence of cognitive disorders, making it hard to describe symptoms, and/or conditions acting as RLS mimics such as drug-induced akatisia, myelopathy, myopathy, neuropathy or vascular claudicatio (45). Patients with comorbidities and polytherapies represent the most complex scenario, as many drugs can exacerbate RLS severity (e.g., antihistamines, selective serotonin reuptake inhibitors). Moreover, reduced iron adsorption and reduced mobility/sarcopenia may worsen pain/sensory disturbance (44).

## RLS, PLM and sleep fragmentation

According to current diagnostic criteria, PLMS are considered to be associated with cortical arousals (the two events are separated by < 0.5 s), regardless of which appears first (29). Around 17–55% of PLMs are associated with arousals, with longer LMs associated with a higher risk and being more disturbing than shorter ones (46). PLMs may also trigger abnormal arousal reactions evoking NREM sleep parasomnia episodes (47) and/or sleep-related epileptic seizures (48).

Sleep macrostructure in PLMD is characterized by a relevant reduction of slow wave sleep (SWS) with parallel increase of lighter sleep stages (49). A pathological increase of microarousals is also typical of this condition, with a rise of CAP subtypes A2 and A3, the more “disturbing” CAP subtypes, that in many cases (around 95%) are strongly synchronized with limb movements (50). CAP has a well-known double significance: exerting a protective and “sleep promoting” function on the descending slopes of the first sleep cycles and a “wake/REM promoting” effect on the ascending slopes of them. The “buffer system” aspect is a main feature of the dynamics of A1 events whilst A2-A3 events are related to true “sleep fragmentation”, the commonest sleep microstructural fluctuation in symptomatic PLMD.

The relationship between PLM and sleep microstructure is not always predictable and, in some cases, the sleeping brain activity can keep its periodic oscillatory pattern, while limb jerks might disappear with medications (dopaminergic drugs) or, vice-versa, LMs can persist with minor impact on brain activity (clonazepam), suggesting a form of complementarity between these medications. Thereafter, the decision to focus on the limb motor phenomena rather than ensuring a more stable sleep continuity, is always a puzzling challenge for the clinician. The combination of both clonazepam and dopaminergic medication, if well-tolerated by patients, is probably the most adequate choice to guarantee a more comprehensive approach to this “multidimensional/multilevel” condition.

So far, the relationship between isolated RLS and sleep fragmentation is still blurry, as most studies analyze sleep texture in patients with coexistent RLS/PLMD. However, RLS alone can provoke chronic sleep onset insomnia and (subsequent) sleep deprivation: the two conditions potentially associated with metabolic impairment (reduction in insulin sensitivity, glucose intolerance), vascular dysfunction (impaired endothelial function and vascular stiffness) and increased sympathetic activity (51). Patients with moderate-to-severe RLS present higher intensity of sympathetic activation following LMs compared to mild RLS, showing around 6-times more episodes per night of movement-associated transient heart rate increase (52).

As the majority of polysomnographic studies so far enrolled patients with overlapping RLS/PLMD, the impact of isolated RLS on sleep microstructure remains to be studied.

The management of RLS focuses on non-pharmacological interventions (sleep hygiene; avoidance of caffeine stimulating beverages, smoking, alcohol and excessive physical activity), reserving chronic medications only for strongly symptomatic patients. RLS drugs include dopaminergic, opioids, alpha-2-ligands, benzodiazepines and iron supplementation. No instrumental finding is required for RLS diagnosis, clinical evaluation is considered sufficient to both diagnosis and to attest the efficacy of medications.

Reasonably, the goal of PLMD and RLS therapy should be to ameliorate subjective symptoms while stabilizing sleep structure and limiting the dangerous autonomic consequences of these conditions (If so, instrumental sleep recording should not be suggested for both diagnosis and follow-up?).

## PLM and RLS pathogenesis

Little is known about the pathophysiological differences between RLS and PLMD, frequently coexisting in the same patients and, sometimes intermingled with other sleep disorders (53, 54). Abnormal threshold arousability has already been hypothesized in RLS patients', presenting unusually high cerebro-spinal fluid (CSF) concentration of the wake-promoting hypocretin-1 (55), increased reciprocal connections between sensory-motor cortices and subcortical nuclei (56) brain iron deficiency and hyperexcitability of the spinal cord (57). On the background of genetic predisposition, various external “acquired” factors may modulate the appearance and severity of the disease (58, 59). Accordingly, RLS is considered a “network circadian disorder” with complex multifactorial pathophysiology. So far, less is known regarding PLMD relationship with individuals' arousal threshold and/or pathophysiological mechanisms. It is assumed that PLMD reflects a supra-tentorial/spinal hyperexcitability, maybe favored by dopaminergic dysfunction (60), however given the complex embedment of the disorder within manifold axes it probably depends on the derailment of multiple circuits.

## PLMs and the human “inner oscillatory rhythm”

This interesting result is in line with previous observations suggesting that PLMs may be part of a series of normal rhythmic oscillatory events with 4–90 s wave-lengths in humans including heart rate, blood pressure, respiration and cerebral blood flow dynamics that may find a comprehensive synthesis in CAP (61, 62). In this framework, PLMs have been interpreted as the result of a gradual decrease of cortical inhibition in the brainstem during sleep, in other words, periodic motor movements would be motor epiphenomena of the intrinsic human oscillatory network. Thus, rather than being responsible for sleep fragmentation themselves, they appear in reaction to internal or external perturbative factors (61). Indeed, PLM semiology is known to vary according to sleep stage, body position and external stimuli (63). The former hypothesis is somehow confirmed by the observation that the time-relationship between micro-arousal and PLMs is not always predictable: arousals may precede or follow leg movements or even occur simultaneously (64); accordingly, PLM may both be the cause or consequence of sleep instability (42).

The amount and periodicity of PLMs are probably modulated by the individual's arousal threshold, and, reasonably, LMs take place when disturbing stimuli are not powerful enough to trigger a complete awakening. PLMs are deeply associated to the sleep capacity to protect itself against arousing stimuli; which is a feature of brain resilience that progressively decreases with age, leading to a “para/physiological” increase of PLMs in the elderly. The reason why some individuals, with similar PLMIs, are or are not affected by PLM-related clinical consequences, is still largely unknown. Sleep might be perturbed by at least three types of “arousal reaction” including: cortical arousal, autonomic arousal and movement arousal, each involved in the fight-or-flight response of the brain toward danger, lastly causing sleep fragmentation. Instead of being classified as a sleep-movement disorder PLMD should rather be considered as an arousal disorder. This question is not purely academic, because if PLMD is an epiphenomenon representative of low arousal threshold, then therapy should focus on stabilizing and consolidating sleep itself rather than working on limiting the motor manifestations.

## PLM/RLS and the autonomic system

Current diagnostic criteria surprisingly disregard the impact of PLM on the autonomic system. A higher PLMI has been associated to a higher risk for atrial fibrillation (65), hypertension (66) and poor cardiovascular outcome (67). The coexistence of PLMD with OSA increases the risk of severe systolic hypertension, important vessel-wall stiffness and systemic inflammation (68), raising the issue on whether PLMD should be considered a cardiovascular risk factor (69). RLS/PLMS patients present decreased parasympathetic tone and an increased sympathetic tone mainly during N2 sleep (70), when PLM index is higher. Autonomic activations with PLMs are detectable even without concomitant arousal reactions (71). LMs with shorter IMI (< 10 s) are associated with stronger cardiac activation and more severe autonomic impact (72). Heart rate variability (HRV) represents an indirect and non-invasive way to measure autonomic nervous system balance (73). RLS patients with a more severe disease phenotype, present stronger sympathetic activation associated with leg movements, a phenomenon which has been suggested as predictive of adverse cardiovascular consequences (52). However, the relationship between RLS and cardiovascular risk is still a matter of intense debate and both longitudinal studies and animal models investigations are desirable (74).

HRV signals are assessed with linear measurements (75). Heart rate spectral analysis leads to the estimation of frequency domain indices including: very low frequency (VLF: 0–0.04 Hz), low frequency (LF: 0.04–0.15 Hz), and high frequency (HF: 0.15–0.4 Hz). Physiologically NREM stages are characterized by a progressive increase of the parasympathetic tone, followed by transient sympathetic predominance during REM sleep. However, NREM sleep stages are not stable; they areinterrupted by phasic EEG activations (including CAP fluctuations, spindles and other electrical phenomena when explored at smaller levels), which are typically associated with concomitant autonomic instability. In detail, during periods of unstable NREM sleep, HRV analysis shows an increase in low frequency and low frequency/high frequency ratio, suggesting a transient shift toward a sympathetic predominance (76). Extensive data have been collected with respect to sleep-related HRV dynamics in patients suffering from insomnia (77), OSA (78) or epilepsy (79). RLS/PLMD has been associated with increase in VLF, LF and LF/HF ratio during all NREM sleep stages (70). More recently, several complex non-linear analyses of HRV, including entropy-driven indices, have been developed to detect subtle changes in the autonomic system dynamics, yielding to better mortality prediction (80). Further studies with either linear or more advanced non-linear measures, exploring the autonomic impact of PLM in both symptomatic and asymptomatic patients are advisable. Given the potential role of the adrenergic system in the RLS/PLMD pathogenesis, some medications acting on the autonomic nervous system have been occasionally tested. In detail, Clonidine, a centrally acting alpha-2 adrenergic agonist, demonstrated a weak effect on the subjective sleep measures, with no relevant variations in the PLM metrics. So far only few controlled studies analyzed the risk/benefit ratio of adopting clonidine for RLS/PLMD (81). Further investigations are required to clarify the role of this molecule in the disease treatment protocols.

## Future directions

There is need for a revision of PLMD diagnostic criteria. The raw cut-off threshold of PLM index >15 events/hours is poorly informative (especially in the elderly) and a more “adaptive” cut-off value, corrected for patients' age, is desirable. Other parameters such as the PI may support a deeper understanding of the condition. Perhaps, different PLM index cut-off values could be set for patients with higher cardiovascular risks or with coexistent sleep disorders potentially worsened by PLM motor manifestations. Similarly, high-risk patients with RLS may deserve a polysomnographic exam to check for coexistent PLMD. Furthermore, a comprehensive measurement of PLM dysfunction inclusive of the evaluation of both autonomic impact and sleep fragmentation, would hopefully help in the identification of patients requiring dedicated medical attention. Finally, polysomnographic studies on “asymptomatic” PLMD are advisable.

## Sleep disordered breathing

Sleep breathing disorders are multi-systemic conditions with widespread impact on cardiovascular health, mental status, metabolic balance, quality of life, daytime sleepiness, sleep depth and stability, circulating inflammatory biomarkers (82, 83).

OSA is characterized by periodic partial or complete obstruction of the upper airways during sleep associated with intermittent oxygen desaturation, hypercapnia and cardiovascular dysfunction. It is highly prevalent in adults (84). Recently, OSA has been associated with pulmonary embolism (85), proliferative retinal diseases (86) and non-alcoholic fatty liver disease (87). Sleep apneas are extremely common (and frequently overlooked) in patients with cardiac arrhythmia (88) and they increase the risk for adverse maternal outcome in pregnancy (89). Children experiencing sleep apnea are at higher risk for cognitive deficit, its reversibility is not completely understood (90). A strong bidirectional relationship between sleep apnea and neurodegenerative diseases, such as Alzheimer's disease (AD), has been demonstrated (91). OSA and AD share a progressive cerebral accumulation of beta-amyloid (Ab) in the framework of chronic low-grade inflammation and oxidative stress. Apnea-related intermittent hypoxia fuels beta-secretase activity, enhancing amyloid synthesis and accelerating AD pathology (92). In addition, sleep fragmentation in OSA patients hampers the paravascular clearance of toxic molecules, such as Ab and tau, through the night-dependent glympathic system (93).

Given the multiplicity and complexity of OSA affecting several organs and systems, its proper management requires the involvement of a multidisciplinary team with strong expertise in the field. The involved specialists should share a common treatment work-flow, tailoring the treatment of the individual patient and taking into account all indirect implications. Untreated sleep apnea may cause dangerous consequences in peripheral organs and the central nervous system (CNS). OSA features vary according to the degree of physical frailty, sometimes leading to a life-thretening vicious cycle. For example, patients with severe OSA and concomitant cardiovascular impairment may experience the overnight development of central-type respiratory events, reflecting functional exhaustion (94). Patients with COPD (chronic obstructive pulmonary disease) experience a higher risk for decompensation during sleep because of their higher inspiratory muscle tone, increased airway resistance and ventilation-perfusion mismatch (aggravated by the supine position) and frequently suffer from a co-existent sleep-breathing disorder including, above all, OSA. This condition, named “overlap syndrome”, is associated with poorer outcome, increased risk for hospitalization, pulmonary hypertension and cardiovascular events (95). Therefore, OSA patients should always be evaluated in their complexity, focusing on coexistent pathologies, which may impact their outcome and prognosis.

## OSA epidemiology

Recently published world-wide investigations, using AASM (American Academy of Sleep Medicine) 2012 diagnostic criteria for OSA (29), estimated that around 1 billion adults (30–69 years) suffer from mild-to-severe obstructive sleep apnea, with higher rates in China, USA, Brazil and India. Nearly 425 million individuals may be affected by moderate-to-severe OSA (AHI, apnea—hypopnea index > 15/h) (96).

The increasing burden of obesity, diabetes, physical inactivity and aging likely support the continuous growth in the global prevalence of the condition.

Notably, a wide community-based longitudinal cohort study in Louisiana (the Bogalusa Study), that followed 844 children through their middle-age for 35 years, confirmed the increased risk for OSA in the adulthood of children experiencing obesity in their younger ages. The longer the duration of obesity, the higher the risk for OSA in the middle age (97). Targeted educational interventions to prevent obesity in children are of paramount importance for blocking the escalation of OSA.

## The pathophysiology of OSA

Those mechanisms favoring the appearance of sleep apneas can be divided to “anatomical factors” and “functional factors”. Besides the well-known role of pharyngeal anatomy and craniofacial factors, additional, non-anatomical features need to be considered in the pathogenesis of OSA (98). In synthesis, main contributors of OSA encompass: oxygen saturation level in restful wakefulness, anatomical predisposition, upper airway reflexes, body position, sleep stages, sleep instability, arousal threshold, loop gain and concomitant sleep disorders. Non-anatomical traits are increasingly recognized as predictors of treatment outcomes including residual apnea and adherence to therapy, and can be targets of treatment. The improved understanding of upper airway neurochemical control is seeing potential application in pharmacotherapy of OSA with norepinephrine re-uptake inhibitors combined with cholinergic antagonists or sedatives.

## Basal oxygen saturation level

The oxygen-hemoglobin curve, representing the proportion of hemoglobin saturated by oxygen, has a well-known sigmoid shape. This means that, moving to the left, the curve becomes steeper and hemoglobin de-oxygenation can occur easily. Each factor promoting this left-side shift (lower PO2%, increased PCO2%, decrease in temperature) will reduce the hemoglobin affinity to oxygen (99). This “left-sided” scenario is typical in patients with chronic respiratory diseases and explains the higher risk for marked and deeper nocturnal desaturation in this group. Basal wakefulness O2% saturation level helps to predict how far the patient is from the critical threshold for hemoglobin desaturation.

## Anatomical predisposition

Anatomical predisposition to upper airway obstruction in OSA patients includes: brevity of the mandible and uvula; enlargement of the tongue, uvula or pharyngeal walls; retropalatal/retroglossal obstruction. Obesity may also promote upper airway collapse due to fat accumulation in the soft tissue. Pharynx in OSA patients is typically smaller in diameter and face greater variations during sleep due to its higher compliance (100). All the listed variables may increase the risk for phasic collapse during nocturnal breathing. The collapsibility of the upper airways during sleep can be quantified and is named Pharyngeal critical closing pressure (Pcrit) (101). Typically, OSA patients differ from healthy sleepers by a greater tendency of upper airway collapse, as mirrored by higher values of Pcrit (~ +5 cmH20). Patients with higher Pcrit values are those who will probably benefit from PAPs therapies (“anatomical predisposition dominant”). Conversely, OSA patients with normal nocturnal Pcrit are probably strongly influenced by non-anatomical factors that should be investigated to tailor the most effective therapy (“non-anatomical predisposition dominant”) (102). As Pcrit measurement is not available everyday, newer non-invasive tolls are needed for categorizing OSA patients with respect to their upper airways' anatomical characteristics.

## Body position

In the last years it has been suggested that overnight rest in clinostatism could lead the fluid shift from the lower part of the body, where the fluid accumulate in the daytime in fluids overload condition, to the upper body compartments “cause of gravity effect, raising the neck circumference during the night, increasing the collapsibility of the upper airways and, in OSA patients, exceeding the critical closing pressure that finally leads to sleep apnea (103). Furthermore, obese patients experience an increased risk for nocturnal sleep breathing disorders at bed rest, due to their reduction in diaphragmatic activity, thoracic compliance and higher risk for air trapping (104).

The fluid shift reduction through salt and fluids restriction, diuretics and physical activity combined with positive airways pressure and oral appliances could improve the airways obstruction and, consequently, the OSA severity.

## Loop gain

Loop gain refers to the reactivity of the respiratory control system to external or internal perturbations. Loop gain is a system composed of three components: (1) plant gain (lungs), (2) time of delay in circulation and (3) controller gain (chemoceptors). Higher loop gain may induce over or undershooting of ventilation to disturbing stimuli (105). Hence, a patient with “high loop gain” reacts massively to minimal perturbations and is at higher risk for iatrogenic central breathing patterns during non-invasive ventilation, while patients with low loop gain, maintain a stable ventilation (106). Although this difference may influence CPAP efficacy and patients' compliance (107), it is hard to measure or even recognize abnormal loop gain in the standard clinical setting. Simple breath-holding maneuvers have been proposed as an indirect index for loop gain estimation: shorter maximal breath-hold duration and larger post-apnea ventilatory response are commonly associated with higher loop gain during sleep (108).

Loop gain can be reduced with O2-therapy, carbonic anhydrase inhibitors, hypnotics or zonisamide (109, 110). Once again, personalized approaches are desirable.

## Sleep stages

Sleep influences OSA dynamics through various modalities. Some patients are affected by a CAP-dominant sleep apnea, presenting significant prevalence of phasic respiratory events during NREM sleep, with a strong temporal correlation with NREM sleep CAP fluctuations (111). In these cases, the sleep clinician should dedicate major efforts in improving (NREM) sleep stability. Conversely, other patients show a relatively stable breathing pattern during NREM sleep and are more affected by the muscle atonia and autonomic chaos typical of REM sleep, the so-called REM dominant OSA. In REM-OSA, upper airways are typically more collapsible, while loop gain is lower and arousal threshold is higher during NREM sleep (112).

## Sleep instability

Sleep apnea can profoundly disturb sleep stability: affected patients typically present lower amounts of stage N3 with a parallel increase of superficial sleep and an impressively high amount of sleep instability, as demonstrated by higher CAP rate. To what extent OSA can hamper sleep continuity strongly depends on disease severity. Indeed, it is well-known that in moderate-to-severe OSA, sleep texture is characterized by a higher percentage of the more disturbing CAP subtypes A2 and A3 (113).

More recently, it has been shown that in mild OSA the CAP subtypes A1 (slow wave arousals) still prevail and “try” to reinforce sleep continuity. Conversely, in moderate-to-severe OSA, the more disturbing/arousing subtypes A2 and A3 prevail and reverse the sleep-stabilizing role of CAP system, evolving into an intrusive mechanism that finally disrupts sleep continuity (114). Different arousal routes/pathways, depending on the underlying severity of body distresses have also been hypothesized. In this framework the hub-region of the pontine parabrachial nucleus can evoke both “milder” arousal reactions and “stronger” arousal reactions, as mirrored by the appearance of K-complex/delta bursts (CAP subtypes A1) rather than higher frequenciy bands activities (alpha/beta bursts), according to singular conditions (115). CAP dynamics may be used to analyze the dynamics of respiratory-related arousals.

## Arousal threshold

The occurrence of transient arousals from sleep after small changes in ventilatory drive is termed “low arousal threshold” and may predispose to obstructive sleep apnea. Not all respiratory events are followed by phasic arousals and, occasionally, arousals occur after the resolution of the sleep apnea (116). Physiologically, the ventilatory drive activates the pharyngeal dilator muscles, preventing sleep apneas. OSA patients with low arousal threshold experience premature arousal intrusion during sleep, increasing the risk for unstable breathing patterns (117). These arousal intrusions can frequently yield to abrupt ventilatory responses with fluctuations in CO2 concentration, lastly fueling the nocturnal instability of respiratory control. Few medications had been proposed to treat OSA in patients with lower arousal threshold (benzodiazepines, Z-drug, trazodone), however their administration might worsen nocturnal hyopxiemia and AHI, as some of them can enhance pharyngeal muscle relaxation and delay arousal, thus worsening hypoxemia. A detailed and personalized evaluation of the risk-benefit ratio is mandatory before trying this strategy.

## Apnea duration

According to current guidelines, a nocturnal breathing event must last at least 10 s or more to be deemed as apneas or hypopneas. Mean apnea duration is a frequently used parameter to estimate respiratory events' length. Debate is still ongoing on whether longer or rather shorter apneas should be considered at higher risk for cardiovascular consequences (118, 119). In their investigation Sarac et al. revealed that morning tiredness, sleep fragmentation, reduced blood oxygenation and hypertension were more frequent and severe in patients with longer apnea duration (119). Longer apneas probably reflect a progressive increase in the arousal threshold, which may partly occur with age, and, in parallel, a delayed response to oxygen desaturation. Conversely, shorter breathing events may rapidly accumulate resulting in higher AHI scores per hour, automatically worsening the severity of the condition.

## Concomitant sleep disorders

Routine clinical practice is often complicated by the overlap of sleep apnea with other sleep disorders (PLMD, NREM sleep parasomnia, epilepsy, RBD, narcolepsy). Coexistent sleep pathologies may lower patients' compliance to OSA treatment, and untreated sleep apneas may trigger abnormal arousals leading to NREM sleep parasomnias or nocturnal seizures (120). The relationship between OSA and epilepsy is a complex and bidirectional one: epileptic patients with untreated OSA experienced more nocturnal seizures and, in drug-resistant epilepsy the prevalence of OSA is estimated around 33% (121, 122). The two conditions share the reduction of REM sleep that had been proposed as a biomarker for epilepsy severity/drug-refractiveness (120, 123). Accordingly, the restoration of REM sleep should be included as one of the main goals of OSA and epilepsy therapies (124). Current recommendations for non-invasive ventilation management in OSA patients state that 4-h of nocturnal CPAP therapy can be considered “enough”. However, as REM sleep physiologically predominates in later parts of the night, we are wondering if this could guarantee sleep and health amelioration in OSA patients.

PLMD may also be associated with phasic respiratory events, mostly hypopneas. These respiration- related movements, according to current guidelines, should not be included in the final computation of AHI index. The two conditions (OSA and PLMD) are modulated by the same permissive “windows” for cerebral and autonomic activation, mirrored by the CAP phases A (motor activation) and B (respiratory inhibition). A recent multicenter randomized controlled trial explored the prevalence of PLMS in a large cohort of 1,105 OSA patients (125). According to their results around 19.7% of OSA patients had PLM index (PLMI) ≥10/h and 14.8% had PLMI ≥ 15/h, with higher risk among the elderly and in those patients taking antidepressants or higher doses of caffeine. Patients with higher PLMI presented worse sleep quality and, notably, 6-months of CPAP therapy did not modulate PLM severity, suggesting that great efforts should be dedicated to the diagnosis and the concurrent management of the condition (123).

Central sleep apnea (CSA) can coexist in OSA patients for several reasons: CSA, especially when a Cheyne Stokes breathing pattern is recognizable, can indicate an underlying cardiovascular disorder (e.g., congestive heart failure); in other cases CSA can be associated to opiod intake, the alternation of hyperventilation and underbreathing with central-type respiratory pattern can appear at very high altitude or be associated to chronic disorders (e.g., end-stage kidney disease). Lastly, CSA can be iatrogenic in OSA patients treated with PAPs devices, the so-called treatment-emergent central sleep apnea (TECSA), which can be associated to various potential mechanisms including ventilatory control instability, low arousal threshold, activation of lung stretch receptors, and prolonged circulation time.

Clinicians must dedicate efforts in the understanding of the simultaneous presence of OSA and CSA, as this binomius can be associated to numerous heterogenous conditions.

## OSA and the autonomic system

OSA patients present nocturnal autonomic dysregulation, which can be worsened by coexistent somatic (e.g., hypertension, diabetes) or psychological (e.g., stress, depression) conditions similarly associated with an altered sympatho-vagal balance (126). Sleep exerts direct influence on vegetatitive dynamics shifting between a parasymphatetic (NREM sleep) and sympathetic (REM sleep) dominance (127). Apneic episodes lead to lung inflation, favoring sympathetic activation, which promotes phasic increase of blood pressure and heart rate. Through a baro-reflex mechanism, the post-apnea re-opening of the upper airways determines a transient withdrawal of sympathetic stimulation (78). HRV is one of the most widely used markers of autonomic functioning. HRV high frequency (HF) power (0.15–0.4 Hz) reflects a vagal dominance, whilst low and very low HRV frequency (0.04–0.15 Hz) are associated with both sympathetic and parasympathetic activation. Sleep apneas are associated with increase in LF and decrease in the complexity of heart rhythm (128).

## OSA complexity and subtypes

The current denomination of OSA is partially misleading, referring, as major determinant of the condition, to the focal and phasic obstruction of the upper airways during sleep. Accordingly, sleep apnea management is commonly attributed to the expertise of pulmonologists or ENT specialists. Although the role of these specialists is beyond questions, the multi-facet aspects must never be neglected. The disease might better be defined by the inclusive term “sleep disordered breathing”, where the complexity of sleep mechanisms are taken into account.

As explained before, sleep apneas and hypopneas can be heterogeneous events. Whereas, the term “phenotype” typically refers to different clinical manifestations, the concept of endotype tries to include more details on the disease pathogenesis. Distinct sleep apnea endotypes have been proposed, depending on numerous features such as anatomical characteristics, arousal threshold and ventilatory control stability (129).

The multinational ESADA (Sleep Apnea Network/European Sleep Apnea Database) group (a pan-European, multi-center research group) dedicated great efforts in the characterization of different OSA clusters (130). Applying the latent class analysis to large data sets they were able to describe eight distinct phenotypes: Four based on gender and four based on a combination of age-bands, BMI (body max index), AHI and burden of comorbidities (see Bailly et al. for details). Once again, the authors emphasized the importance of a tailored, gender-based and comorbidities-based evaluation of OSA patients.

OSA can also be “sub-classified” according to symptom-severity or to coexistent cardiovascular comordities (131). The role of comorbidities in the “OSA scenario” (named CoSA or Comorbidities of Sleep Apnea) have recently been proposed to stratify patients according to their global clinical burden (132). It seems that most OSA patients suffer from at least 3–4 comorbidities and, the number of comorbidities may help to predict outcome and mortality.

Disregarding the complexity of the disease raises the risk of oversimplifiing pathology, overlooking important features, and lastly, augmenting the risk of treatment failures.

## OSA therapies: A multidimensional approach

OSA complexity also reflects in its management. As for many other chronic conditions it is key to guarantee periodic patients' re-evaluation, essential to guide a tailor approach according to individuals' condition, that might largely change with variations in body weight, cardio-vascular comorbidities, medications. This also requires a multidimensional approach, with a dialogue between numerous specialists: neurologists, pneumologist, otolaryngologist, maxillofacial surgereons, cardiologists and endocrinologists.

OSA patients should always be encourage to pursue on some life-style changes (physical exercise, weight loss, alcohol and smoke avoidance) and must pay attention to sleep hygiene. The condition then might be treated using positive airways pressure (including auto titrating-CPAP, fixed CPAP, BPAP or ASV in selected cases), oral devices, surgical removal of tissue (commonly uvulopalatopharyngoplasty, nasal surgery), hypoglossal nerve stimulation, maxillomandibular advancement or bariatric surgery. Few medications can be adopted in OSA treatment, including the recently approved Solriamfetol to tackle with residual excessive daytime sleepiness, as detailed below. Some other drugs can worsen the condition (e.g., sedative medications) and therefore should be discontinuated, when possible according to patients' condition.

## OSA severity: Time to move beyond the AHI

Currently, sleep apnea diagnosis relies on simplified data such as AHI, with no consideration of the direct or indirect signs of sleep fragmentation, comorbidities and autonomic consequences each infuencing outcome.

In recent years worldwide, major research efforts have been cowed out to “move OSA beyond the AHI index”.

Respiratory events may reflect different pathogenetic mechanisms and variable clinical implications: central periodic breathing is typically less intrusive, with lower autonomic stress. Obstructive events are typically abrupt and disturbing, leading to an increase of negative intra-thoracic pressure and powerful autonomic activation. Respiratory events are usually longer during REM sleep, with more severe and longer lasting desaturations, while mixed apneas may mirror the fatigue of the cardio-respiratory system (133). Central sleep apneas must be distinguished from the “treatment-emergent central sleep apnea”, appearing during CPAP therapy (134). Finally, patients with pre-existent pulmonary diseases will experience deeper oxygen desaturation in relation to milder respiratory events, due to their lower basal SatO2% level. Intuitively, such a complex scenario cannot be summarized in a raw index such as AHI.

Recently some novel biomarkers for OSA severity have been proposed including: AHI in REM sleep stage (REM-AHI), nocturnal hypoxic burden, the pulse rate response to apneas/hypopneas (ΔHR) and others (135–137). The hypoxic burden, calculated as the oxygen desaturation “area under the curve” in association with individual apneas and hypopneas, appears to associate with incident heart failure. Thus, it could be a useful marker identifying patients with higher cardiovascular risk (137).

Recent proteomic investigation reveals distinct protein signature in obstructive and central sleep apnea: OSA presenting disturbances in the expression of various proteins involved in coagulation, inflammation, growth factors and hemostasis; central sleep apnea associating with an abnormal proteomic profile in molecules involved in the balance of the pre-Bötzinger complex (a cluster of interneurons in the medulla), essential for the generation of the spontaneous respiratory drive in mammals (138). Metabolomic and microbioma investigations provided confirmations for these findings highlighting the roles of certain metabolites related to fatty acid, carbohydrate and amino acid metabolism in the pathophysiology and cardiovascular complications of OSA (139).

In summary, AHI oversimplifies the characterization of OSA severity and could not be used alone to predict patients prognosis.

## The importance of the follow-up plans

OSA severity typically progresses with patients' aging. Compliance may decline with time and the adherence to therapy is the major limitation of CPAP therapy. A minimum of 4 h per night for at least 70% of nights is considered essential to ensure therapeutic efficacy of non-invasive ventilation. There is a dose-response relationship between CPAP utilization and benefits on symptoms (daytime sleepiness) and other health consequences (hypertension) (140). As REM sleep typically prevails during the second part of the night, the 4-hours' cut-off of nocturnal ventilation may not be entirely sufficient to guarantee a proper management of OSA as discussed above. REM sleep is frequently curtailed in OSA patients and there is a rapid REM-rebound after the introduction of CPAP (or BIPAP) therapy. It is likely, that for preserving REM sleep integrity, a longer nocturnal PAP-use should be required.

Regular follow-up visits to guarantee patients' compliance are also of pivotal importance in the clinical assessment of OSA patients. As already outlined, OSA is a chronic and dynamic condition, warranting periodic re-evaluation. For exemple, the nocturnal breathing pattern might change after acute or chronic cardiovascular events, with the appearance of central-type/mixed-type of events, imposing variations of PAPs parameters and, sometimes, the shift from a CPAP to an adaptive servo-ventilation (ASV) machine or to a BiLevel positive airway pressure (BiLevel) therapy. The activity and involvement of a sleep-team with doctors and technicians is necessary. CPAP prescription is the very first therapeutic acion of a long-lasting clinical dialogue between sleep centers and OSA patients.

## OSA and hypersomnolence

The reason why EDS is common in patients with severe OSA is still under debate. In 1993, a higher prevalence of hypersomnolence was found in individuals with an AHI ≥ 5 compared to those with AHI < 5, and, in individuals with an AHI < 5, a higher prevalence was found than in habitual snorers (141).

In a recent debate on the relation between sleep fragmentation and hypersomnolence in OSA, Punjabi and Lim (142) declared that arousals are the causative elements for daytime sleepiness in sleep apnea. Airway collapse or closure can lead to hypercapnia and increase the breathing work, which may contribute to impairment in daytime alertness. However, both are likely to mediate their effects through arousals from sleep and disruption of sleep continuity. Murine models have shown that exposure to long-term intermittent hypoxia, in the absence of sleep fragmentation, results in oxidative neural injury to wake promoting neurons and this injury could also be responsible for daytime sleepiness (143). With such evidence, it is reasonable to conclude that both sleep fragmentation and intermittent hypoxemia contribute to the development of daytime sleepiness in sleep apnea.

In contrast Gold and Gold (144) stated that the sleep fragmentation paradigm accounts for only some of the hypersomnolence identified in OSA patients: at any level of AHI, snorers are generally more somnolent than non-snorers. In other words, snoring frequency is an independent correlate of hypersomnolence, unrelated to sleep fragmentation by arousals. Both AHI and snoring frequency reflect a single underlying pathophysiologic factor, inspiratory flow limitation (IFL), which is expressed both in the OSA subgroup and in the snoring subgroup. Combining the two groups into one population there is only one fundamental predictor: IFL. Investigators automatically equate increasing AHI with increasing sleep fragmentation rather than choosing to equate it with increasing time spent in IFL. If the most direct causes of sleep fragmentation are arousals, wouldn't one expect to easily demonstrate a correlation between hypersomnolence and arousal frequency?

Punjabi and Lim (139) reply that scoring of arousals based on conventional criteria completely neglects an entire family of subcortical physiological events that reflect central nervous system activation such as the autonomic surges and the appearance of delta bursts or K-complexes in the electroencephalogram (EEG). To focus only on arousals is akin to only seeing the tip of the iceberg and neglecting the complexity of events that characterize sleep instability. Because sleep fragmentation is commonly operationalized using arousals, the omission of other, more sensitive, measures of sleep state instability (CAP) could certainly explain the poor association between arousal frequency and daytime sleepiness in sleep apnea. In large population based samples, individuals with OSA can be hypersomnolent and yet not have lighter sleep than those without hypersomnolence. Comparing polysomnographic parameters between sleepy and non-sleepy subjects with moderate to severe OSA (*n* = 1,115) Kapur et al. showed identical total sleep time, sleep efficiency, arousal index and sleep stage distribution in the sleepy and non-sleepy groups (145). In 2007, Guilleminault et al. (146) have showed that patients with UARS (upper airway resistance syndrome) have higher EEG arousal indexes and important NREM sleep disturbances that correlate with subjective symptoms of sleepiness and fatigue. These disturbances are identifiable with sensitive measures such as CAP analysis but not with traditional diagnostic and scoring systems. All 30 patients with UARS reported chronic fatigue, 28 reported non-refreshing sleep, 26 reported disrupted nocturnal sleep, 17 reported morning headache, and 29 reported daytime performance impairment. The mean ESS was 8.5 in UARS patients compared to four in healthy controls. According to the UARS criteria outlined by Bao et al. (147), syptomatic subjects must have an apnea-hypopnea index of 5 or less, a minimum oxygen saturation >92% during nocturnal sleep and mild upper airway collapsibility. In 2018, Korkmaz et al. (148) divided 38 male patients with moderate to severe OSA into two subgroups, with and without daytime sleepiness (ES) based on the ESS. While there was no difference in clinical characteristics and sleep macrostructure between the two groups; OSA patients with ES had increased CAP measures compared to those without ES: more disruption of sleep continuity is associated with more ES. In 20 male individuals with OSA, the occurrence of CAP A-phases (cortical activation) and PWA drops (below 30%) was quantified in relation to apneas, hypopneas or flow limitation events. A dual response (A-phase associated with a PWA drop) was the most frequent response for all types of respiratory events, with a progressive reduction from apneas to hypopneas and IFL. The highly significant correlation in total sleep time (*r* = 0.9351; *P* < 0.0001) between respiratory events combined with A-phases and respiratory events combined with PWA drops was confirmed both in NREM (*r* = 0.9622; *P* < 0.0001) and REM sleep (*r* = 0.7162; *P* < 0.0006). In conclusion, a dual cortical and autonomic activation is the most common manifestation at the recovery of airway patency. These findings indicate a close temporal connection between events upstairs (EEG) and downstairs (autonomic system). The problem is the tools we use to investigate the body language during sleep. Vegetative microevents (apneas, hypopneas, IFL, PWA drops) require a repertoire of neurophysiological microevents (CAP phase A and B). EEG arousals can provide useful information but they carry two major inconsistencies: (1) they are the iceberg tips of a wide spectrum of activation features which include also K-compelxes and delta bursts; (2) they offer a static and limited picture (< 15 s) of a more dynamic condition of sleep instability (CAP sequences) which can last several minutes and translate the alternating double-face oscillation of activation (phase A) and inhibition (phase B).

In conclusion, in OSA patients, unstable sleep and the multifold features of EEG activation (A phases of CAP) play a topical role in ES, while IFL reflects milder disruption of sleep consolidation and continuity. However, ES is a multifactorial condition, which can be independent of AHI, arousal frequency or CAP parameters. OSA is not a prerequisite of hypersomnolence, which, it can be triggered by a trivial drop of blood sugar levels. In narcoleptic patients, ES is attributed to reduced amounts of hypocretin in CSF but whether hypersomnolence is also fueled by nocturnal sleep disruption and a compared to healthy controls remains an open question. A patient with Kleine-Levin syndrome showed small intenstine overgrowth and the recurrent episodes of ES vanished after regular treatment with local-acting antibiotics (149). These considerations do not clarify the puzzling nature of hypersomnolence, but certainly indicate that the pathways pursued so far by the community of sleep experts need novel and alternative routes.

## OSA and COVID-19

Sleep breathing disorders (SBD) are considered risk factors for COVID-19 severity, with prevalence reaching up to 28% of patients in ICU cohort (150).

Untreated SBD, especially when associated with severe nocturnal hypoxemia, may lead to chronic oxydative and inflammatory damage, increases the risk for acute vascular disease and might contribute to worsen the clinical outcome in post-COVID cohort (151).

There is an impressive clinical overlap between subjects affected by severe COVID-19 disease and OSA patients (middle-aged, obese male patients affected by metabolic syndrome).

Although epidemiologically the two conditions are associated, it is still unclear whether the utilization of non-invasive ventilation devices (PAPs) in moderate-to-severe OSA may or may not prevent from COVID-19 life-thretening consequences. The issue is not trivial as in many countries, during the peak of the pandemia, the utilization of PAPs was discouraged by numerous international societies due to concerns on virus diffusion. Furthermore, the outbreak reduced the connection between patients and healthcare providers, reducing OSA patients' compliance to therapy. However, PAP devices are known to promote numerous cardiovascular protective effects, reduce hypercoagulability decreasing platelet activation, clot strength, hematocrit and lowering blood viscosity while increasing functional residual capacity and thereby improving gas exchange.

Further studies are advisable to assess the relationship between OSA, COVID-19 and well-performed PAP therapy (152).

## Future directions

Sleep teams should evaluate OSA patients in their complexity, trying to embed them into phenotypes and endotypes for tailored therapy. A dialogue between all involved specialists is essential to guarantee effective management, monitoring co-existent or developing systemic and sleep-related comorbidities. Regular follow-up is essential, for ensuring compliance and update the dynamics of the condition.

## DOA: NREM parasomnias

DOA are prevalent conditions: they affect up to 13–39% of children and around 1.6–4% of adults, where they can appear “*de novo*” or persist since childhood (153, 154).

Whereas, NREM parasomnias are usually harmless during childhood, violent behaviors, with even forensic implications, can occur in their adult counterparts (155–160).

Sleep deprivation as well as any factors leading to sleep fragmentation (*via* increasing the homeostatic pressure) are considered the most important triggers for these conditions.

DOA episodes typically start with an abrupt arousal from NREM sleep during the first sleep cycle, when it turns from the descending to the ascending slope. As a consequence, the frontal motor regions partially arise, while the dorso-frontal areas continue sleeping, yielding to a dissociated sleep/wake condition. Several studies have evidenced this brain state dissociation during NREM parasomnias (161–163).

## DOA therapy

DOA should be treated focusing on comorbid sleep disorders and eliminating potential external triggers (e.g., factors increasing sleep fragmentation such as noise or pain, or factors augmenting sleep inertia such as circadian misalignement). Numerous drugs have also been associated with the condition (sodium oxybate; various antidepressants including amitriptyline and buproprion; antipsychotics drugs; metoprolol and topiramate) and, therefore, they should be discontinuated when possible. Clinicians must always educate patients on the importance of a safety bedroom environment, in order to reduce the risk for sleep-related injuries. Usually DOA patients do not require specific therapies. However, when clinically necessary (high frequency of episodes, sleep-related injuries, excessive daytime sleepiness, psychological consequences), few approaches can be pursued, either with cognitive-behavioral strategies or using pharmacotherapy. Clonazepam is still the most commonly adopted first-line agent, alternatively some antidepressant medication can be tried, preferring those with a strong serotoninergic effect (e.g., paroxetine) (164).

## Sleep-related hypermotor epilepsy, the epileptic counterpart of DOA

SHE incorporates any sleep related epilepsy with hypermotor seizures, irrespectively of etiology or types of movements. Most SHE cases are sporadic, while a minority has an autosomal dominant genetic background, associated gain-of function mutations, the best known one affectingthe nicotinic acetylcholine receptor (nAChR) gene subunits (165, 166). The electro-clinical symptoms do not permit a clear-cut discrimination between the genetic and sporadic forms (167).

SHE can be particularly challenging for the clinician as interictal epileptiform discharges (IEDs) hardly occur either in wakefulness or during sleep, detected in < 50% of patients (168). Movement artifacts can cover IEDs and ictal EEGs and the scalp EEG may not be able to reveal a deep-located seizure onset zone. Conversely, when SHE is associated with a structural etiology, abundant IEDs may emerge easing the diagnostic work-up; this is typically the case with focal cortical dysplasias (169).

In 2017, Licchetta et al. (170) studied the etiology and long-term outcome in a large cohort of SHE patients. In this analysis SHE was sporadic in 86% of cases, familial in 14% and associated with structural etiology in 17% of cases. At 30-years of follow-up the overall remission rate was 28.4%.

There is an interesting overlap between NREM parasomnias and SHE. Literature data highlighted frequent positive family histories for both epilepsy and parasomnias in SHE and DOA patients (155, 168), and the lifetime prevalence of DOA episodes seems higher among SHE patients' relatives compared to healthy subjects' families.

## SHE therapy

SHE cases associated with cortical malformations should always be directed to a pre-surgical evaluation. Genetic etiology does not represent a contraindication for surgery. SHE cases with unknown origin are usually treated pharmacologically. Low doses of carbamazepine still represent the most commonly adopted pharmacological strategy for SHE patients. As an alternative, oxcarbazepine, lacosamide or other antiepileptic medications can be used, especially in cases non-respondent to carbamazepine. Fenofibrate, a lipid-modulating drug acting as an agonist of peroxisome proliferator-activated receptor-alpha, demonstrated some benefits in drug-resistant cases (171).

## Commonalities between DOA and SHE

The clinical similarities and differences between DOA and SHE were well shown by the Derry study (172) comparing the semiology of 63 SHE seizures with 57 DOA episodes based on video-EEG monitoring. Three main patterns appear; usually in mixed forms: (1) Simple arousal behavior: eye opening, head elevation, staring, face rubbing, yawning, stretching, moaning, and mumbling; (2) Non-agitated motor behavior: sitting forward, manipulation of nearby objects with a passive or perplexed facial expression. Coherent speech-fragments were frequent; (3) Distressed emotional behavior: fear marked by facial expression or speech content; screaming, violent movements. Most of parasomnia-related events and nocturnal seizures began with an arousal, but only 25% of DOA vs. 88% of seizure terminated in waking. SHE episodes ranged from minor motor events, paroxysmal arousals and major attacks.

The semiological overlaps and major differences between DOA and SHE have been explored by Loddo et al. recently (173). The most important finding of these studies, is the semiological similarity between the two groups, both presenting with an increasing complexity in terms of motor pattern, raising the possibility of a continuum between the two conditions.

The well-known major differences are represented by the number of episodes/night (multiple in SHE vs. one or few in DOA); stereotypy, dystonia and hypermotor features, which are distinctive for SHE; a stronger correlation with external triggers (e.g., noise) in DOA. Both types of manifestations link to NREM sleep microarousals (174). This is in accordance with the finding that DOA episodes occur at the turning point of the first NREM sleep cycle, where deepening turns to rising, while seizures link to CAP A phase, paralleling the overnight homeostatic decay. This implies, that both DOA and SHE strongly correlate with the first sleep cycle occurring within a few hours after sleep initiation.

Notably, both DOA and SHE share a relevant increase of CAP fluctuations: in the two conditions there is a significant increase of CAP subtypes A1, whose appearance frequently precidict the beginning of an (either epileptic or parasomniac) episode. Indeed CAP A1 can be described as permissive framework for highly synchronized nocturnal episodes.

**Figure 3:**
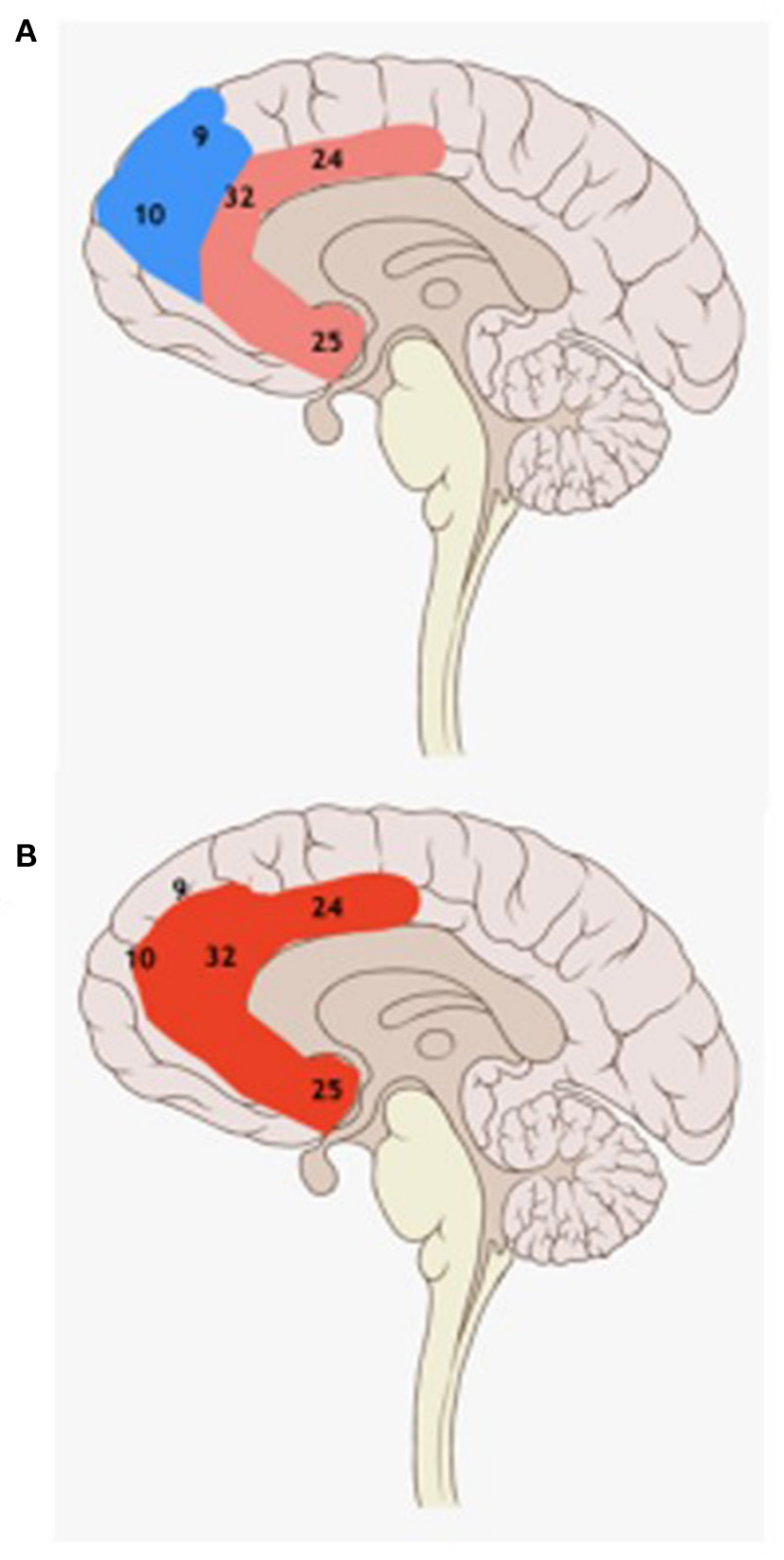
Schematic drawing of the medial brain surface with Brodmann areas. Pink: prefrontal lobe, light red: the pyramidal system **(A)**; red: the anterior cingulate area (Br. 24, 25, 32) **(B)**. There is a dissociation during DOA episodes between the blue fronto-dorsal cortex (in partial sleep) and the red anterior cingulate cortex (in partial wakefulness). SHE seizure onset zones of successfully operated SHE patients frequently overlap with DOA episodes' activated red anterior cingulate area.

In summary, the two conditions share similar mechanisms (disordered arousal during NREM sleep stages), involving the derailment of sleep-related brain circuits yielding to the development of similar hypermotor symptoms, and manifest intra-individual and family overlaps (170, 175–177) (Figure 3).

## Open questions

There are still various unanswered questions regarding both DOA and SHE.

First of all, the definition of SHE has loose boundaries: it involves several types of etiologies, the EEG is highly dishomogeneous and the level of sleep relatedness also appears variable: the spikes of Taylor type focal cortical dysplasia link strongly to sleep, while other forms are less sleep-related (178). Thus, a doubt has persisted on the uniformity of the SHE groups (120, 176, 179).

Also, the definition of “sleep relatedness” needs an extension. Traditionally, only seizures were taken into account, classifying SHE into the sleep-related group. However, the significance of IEDs embedment within sleep texture seems similarly important. Following such approach MTLE (high level of spiking in sleep), and/or absences (rhythmic spike-waves promoted by sleepiness/sleep transition into NREM sleep) can be considered sleep-related epilepsies.

The relationship between arousals and sleep in DOA and SHE is still a matter of debate: two recent studies (120, 159) highlighted the role of the fascinating clash between sleep and arousal forces in triggering seizures/events.

Congruent data support an augmented propensity toward arousal in both DOA episodes and seizures.

In deep NREM sleep stages, arousals can hardly evoke full awakening. In DOA and SHE, partial arousals may result in “dissociated” states. DOA episodes occur when strong ultradian forces turn deep NREM sleep toward arousal/sleep lightening, while seizures seem to link to less intense arousal-fluctuations. Thus, a stronger clash of sleep-wake forces is needed for DOA episodes to occur, whereas SHE can develop more frequently at the less intense collisions of sleep protecting and sleep promoting forces represented by CAP fluctuations.

Therefore, it is evident that the two conditions share some fascinating commonalities.

It is also interesting that there is a partial topographic overlap between brain areas involved in the DOA pathogenesis and the seizure-onset zones in successfully operated SHE patients.

Based on SPECT and electrophysiology studies on DOA patients (162–165, 180) sleep-dissociation consistently localizes to the cingulate cortex (partially awake) and the fronto-dorsal cortex (partially sleeping). Those regions may have different “arousability” thresholds (Figure 3).

Epilepsy surgery experiences show that SHE seizures originate from the anterior cingulum (181–184) and the prefrontal-medial cortex in a higher proportion of cases (185).

The involvement of similar brain areas in seizures and DOA episodes confirms the analogies in their pathophysiological mechanisms and may emphasize the key role of the medio-fronto-cingular cortex for emotional arousal from NREM sleep.

## Theories about the origin of hypermotor phenomena in DOA and SHE

The origin of ictal hypermotor phenomena and panic/agitated DOA behaviors is still a matter of debate; it is historically known that the frontal lobe is likely involved. It has been supposed that SHE seizures may result from the activation of subcortical generators: when the physiological top-down frontal inhibition due to seizure activity ceases, those atavistic (animal or infant) movements arise and may disengage as described in the “music box” approach of Tassinari (186). The dissociation concept is even better seen in DOA (162, 163): the cingulate cortex is active while the fronto-dorsal cognitive field sleeps. The hypermotor semiology may have a combined, evolutionist reason: due to the evolving arousal, the half-sleeping individual experiences threat, developing an alarm reaction with the activation of innate ancient hypermotor patterns (187).

The movement patterns and complexities in SHE and DOA are also variable, reflecting localization, spread and propagation-speed of this phenomena from or to eloquent brain regions (188, 189).

## Future directions

Advanced electrophysiological studies on polysomnographic recording and computer-aided neuroimaging protocols (morphometric brain MRI, EEG-fMRI and other techniques) might provide some novel insights into the mechanisms of DOA and SHE pathogenesis, to assess the magnitude of similarities between the two conditions. This may support a deeper understanding of the concealed link between these two sleep-related hypermotor conditions.

## REM parasomnias

REM parasomnias include several peculiar conditions; REM sleep dissociation symptoms as REM sleep behavior disorder (RBD), recurrent isolated sleep paralysis, nightmare disorder; and other variable syndromes e.g., sleep related painful erection (29, 190). In REM parasomnias an admixture of wakefulness and REM sleep has been theorized, as explained below. We overview REM parasomnias focusing on REM sleep behavior disorder (RBD).

## Short physiology background

The pattern of REM sleep, also called “paradoxical sleep” is made by the combination of striated muscle atonia, rapid eye movements and a wakefulness-like EEG background activity. The EEG is strongly desynchronized, “saw tooth” waves (STW) and rhythmic hippocampal slow waves had been recorded (191, 192).

**Figure 4:**
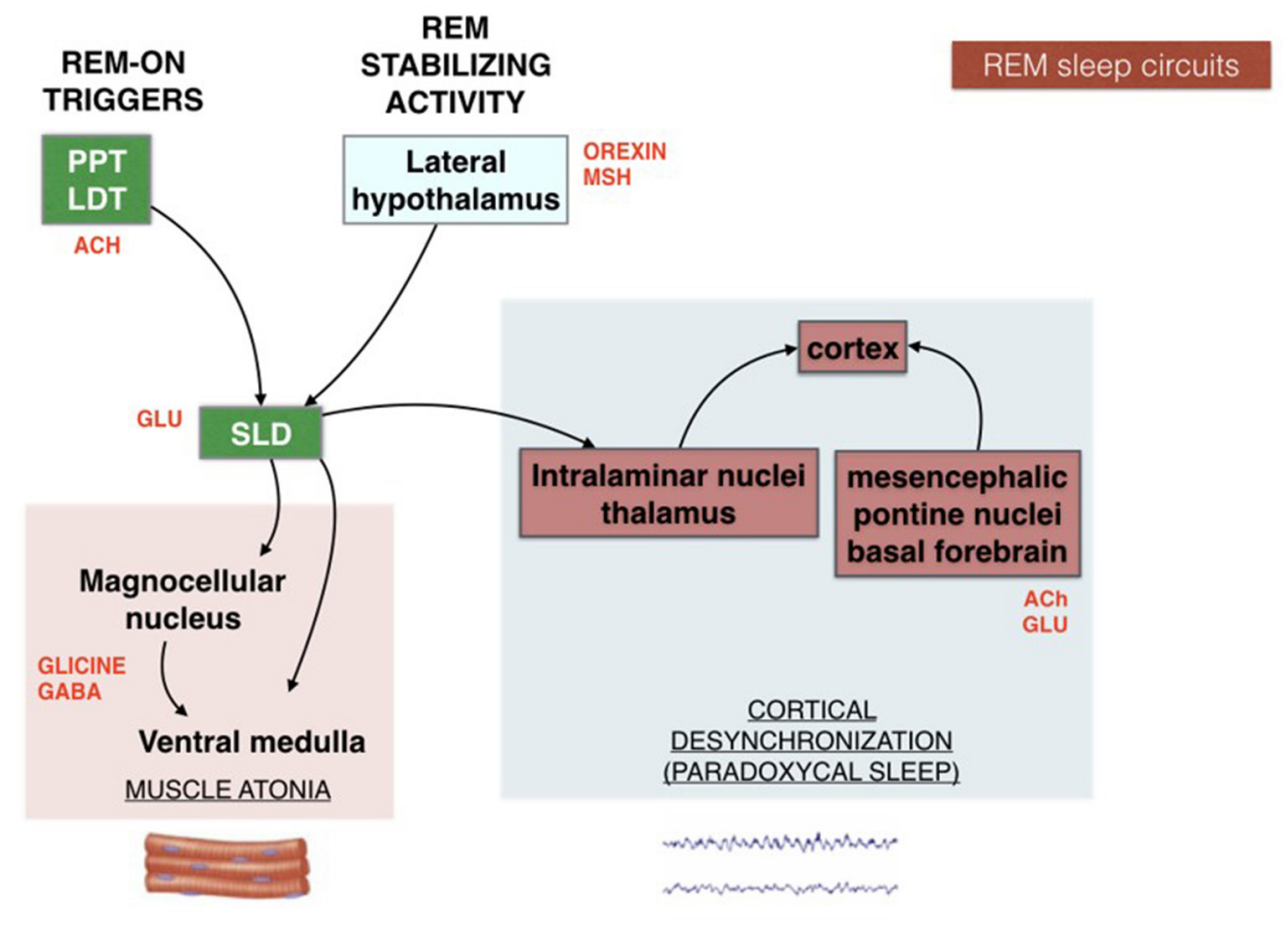
Schematic representation of REM sleep circuits.

Jouvet's pioneer cat-brain trans-section experiments have identified the neural circuitry of REM sleep in the brainstem, mainly in the dorso-rostral pons. A twin REM on/off system regulates REM sleep components through multiple ascending and descending trajectories (193–198) (Figure 4).

REM sleep is believed to be strongly linked with mood regulation, creative problem solving and emotional memory consolidation (199, 200). Dream reports can be elicited after awakening from any sleep stages; however, REM sleep is considered the “dream-phase” with longer dreams and bizarre dream-contents (201).

**Figure 5:**
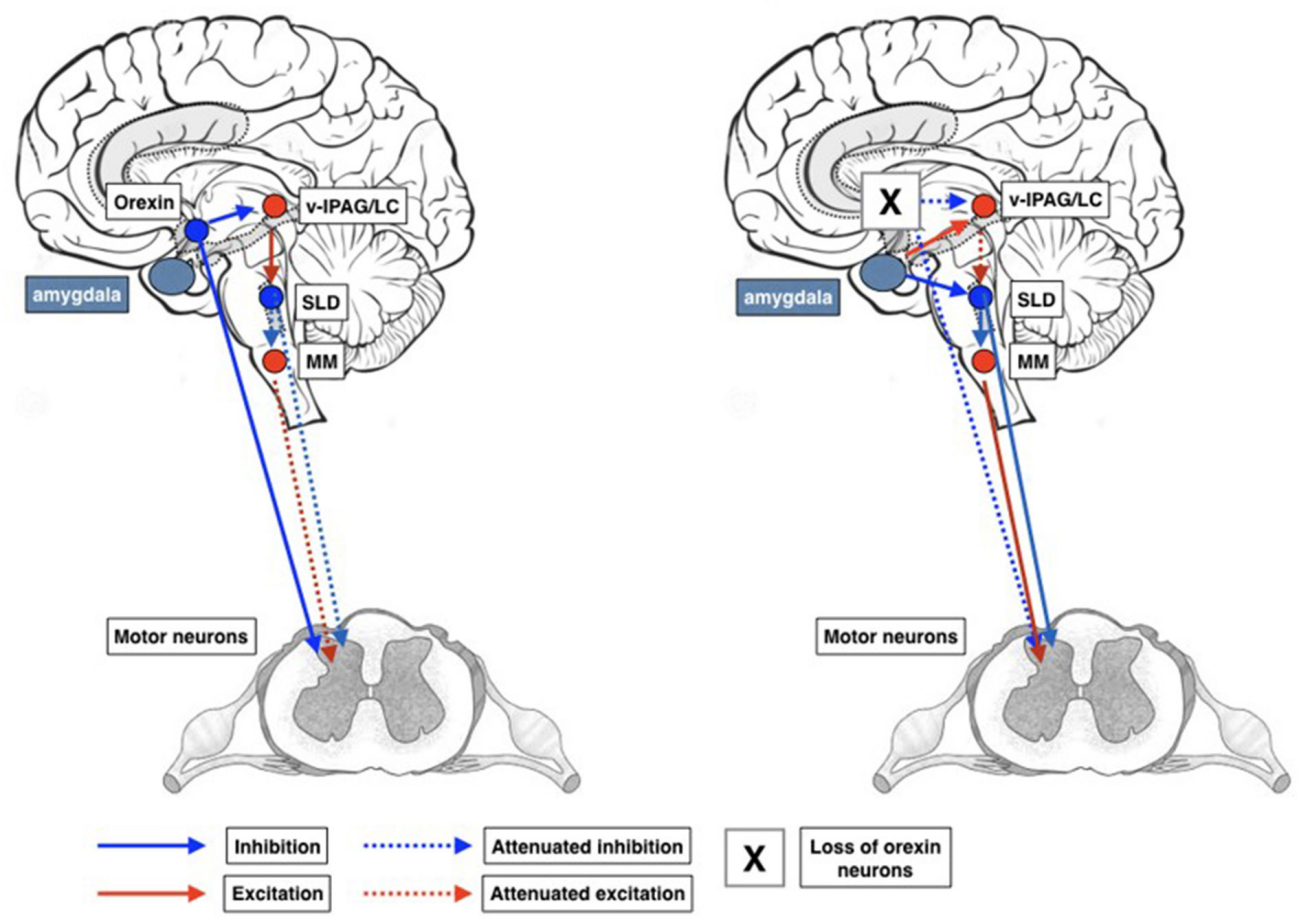
Schematic representation of circuits involved in the development of cataplexy. Inappropriate activation of the REM sleep atonia circuitry during wakefulness is thought to produce cataplexy. Glutamatergic REM-active SLD neurons trigger the paralysis of REM sleep *via* stimulation of the GABAergic/glycinergic cells in the MM. These MM neurons send inhibitory projections to skeletal motor neurons. Under normal conditions, strong positive emotions are processed *via* GABAergic neurons of the CeA, which then inhibit cells in the LC and vlPAG. However, in the absence of the LH hypocretinergic neurons in cataplexy, this inhibition fails, so the REM sleep atonia circuit is released from inhibition and triggers muscle paralysis while the individual remains conscious. The inhibition of LC neurons during cataplexy removes noradrenergic inputs to motoneurons, thereby enhancing the muscle paralysis of cataplexy. CeA, central nucleus of the amygdala; GABA, γ-aminobutyric acid; LC, locus coeruleus; LH, lateral hypothalamus; MM, ventral medial medulla; SubC, subcoeruleus; vlPAG, ventrolateral periaqueductal gray; MNs, motoneurons.

While the low level of acetylcholine in NREM sleep favors the communication of the dorsolateral prefrontal cortex and the hippocampus sustaining the transfer and consolidation of declarative memory traces (202), the high level of acetylcholine in REM sleep promotes an “emotion-driven memory-processing” involving the amygdala, the anterior cingulate and medial prefrontal cortices (203) (Figure 5). The consolidation of fear memories during REM sleep possibly contributes to post-traumatic stress disorder (PTSD) (204). Different abnormalities of REM-sleep e.g., shorter latency, increased REM-sleep density and fragmentation, may participate in additional neuropsychiatric conditions (205).

Muscle atonia protects the sleeper from “acting out” his/her dreams, and the suppression of motor activities can outweigh certain potentially sleep-disruptive stimuli. In this regard, REM-dependent muscle atonia can be deemed a sentinel of sleep resilience (206).

## Regulation of REM sleep

The key members of the REM-related atonia network are the sublatero-dorsal nucleus (SLD) and the noradrenergic pre-coeruleus region (PC) (207) (Figure 4).

The glutamatergic activation of REM-on neurons in the ventral SLD (208) mediates REM motor atonia through two redundant trajectories: recruiting glycinergic interneurons in the spinal ventral horn and GABAergic/glycinergic neurons in the ventro-medial medulla; inhibiting spinal ventral horn motor neurons in both ways.

A direct noradrenergic pathway links the spinal motoneurons with the locus coeruleus, and a serotonergic one with the dorsal raphe (209) both inhibiting the REM-atonia generation of the SLD (Figure 5).

## REM sleep parasomnias as REM dissociation phenomena

### Sleep paralysis

Sleep paralysis has been described in 1664 (210). Over the centuries, it has often been attributed to the presence of “evil”: demons, the old hag in Shakespeare's Romeo and Juliet…. In this frightening state the affected person cannot move or speak experiencing minutes' lasting paralysis and chest pressure, is unable to call, experience suffocating sensations—something is sitting on his/her chest—or even feeling outside his/her own body. Sleep paralysis resolves spontaneously and can occur as part of the narcoleptic tetrad or solely.

Polymorphisms in the PER2 (Period Circadian Regulator 2) gene, a component of the circadian clock mechanism, have been associated with a genetic predisposition to sleep paralysis (211). Multimodal hallucinations and lucid dreaming co-occur in 75% of cases (212, 213).

Antidepressants (Escitalopam, Venlafaxine) have been suggested for treating the most disturbing cases.

## REM sleep behavior disorder: Missing or fragmented REM muscle atonia allows dream enactment

RBD has emerged out of the big bunch of night-time confusional states and violent activities, the latter reported by 1.7% of the population (214). Firstly described in 1986 (215), Schenck et al. characterized it as a manifestation where the patient “enacts” his/her dreams due to lack of muscle atony during REM sleep (RSWA - REM sleep without atonia).

The diagnostic criteria of RBD are the followings: (1) Repeated episodes of sleep-related vocalization and/or complex motor behaviors. (2) These behaviors are documented by polysomnography to occur during REM sleep or, based on clinical history of dream enactment, are presumed to occur during REM sleep. (3) Polysomnographic recording demonstrates RSWA. (4) The disturbance is not better explained by another sleep or mental disorder, medication or substance abuse (216).

In recent years it has been demonstrated that RBD affects sleep in all aspects: sleep instability in NREM sleep, measured with CAP metrics, is significantly reduced compared to age-matched healthy controls. This information can also provide some prognostic knowledge as a CAP rate < 32.9% signalizes a higher risk and a shorter latency for conversion into a syncleinopathy (217).

RBD is categorized as “idiopathic”, or rather “isolated” when standing alone; and symptomatic or rather “combined” when associated with other neurological disorders. Those combined forms may be iatrogenic related to drug use; may associate to neurodegenerative diseases especially α-synucleinopathies, as well as to tauopathies, TDP (TAR DNA binding protein) 43-pathies; to narcolepsy and to any dysfunction of the REM sleep network (218).

The prevalence of isolated RBD is ~0.38–2% in the population >60 years-old (219) and 5–13% in older community-dwellers doubly affecting men (219). A 1:1 gender ratio has been reported in younger groups (220). There is a high rate of autoimmune comorbidity in women affected (221).

## RBD clinical features

*S*udden, vehement and fragmentary movements, shouts out of sleep are key features of RBD. Injuries are frequent; caused by the patient falling out of bed or the bed partner being attacked by the half-sleeping individual enacting horrifying dreams that are remembered after (222). RBD episodes favor the second half of the night - the period of REM sleep dominance; in contrast to NREM parasomnia episodes emerging in the early hours of night sleep.

Those affected, need to be informed about the risk of injuries. Melatonin 3–12 mg or clonazepam 0.5–2.0 mg may help. Donepezil and Vortioxetin have been recently proposed as additional treatment options (223, 224). Concomitant aggravating factors must be corrected (see below: RBD and antidepressants).

## RBD etiologies

Any etiology impairing the complex REM atonia network may cause RSWA/RBD. The duration (transitory or chronic) and associated features depend on the origin of the brainstem dysfunction (drug, hypoxia e.g., obstructive sleep apnea) or lesion (neurodegenerative, inflammatory etc.).

## RBD and neurodegenerative conditions

There is a striking specificity of RBD converting to a synucleinopathy as Parkinson's disease (PD), diffuse Lewy body disease (DLB) or multisystemic atrophy (MSA), while the disorder links to tauopathies and TDP 43-pathies as Alzheimer's disease (AD), amyotrophic lateral sclerosis (ALS) and Huntington's disease (HD), Guadalopean Parkinsonism and progressive supranuclear palsy (PSP) as well (219–221).

RBD may precede any overt signs of these neurodegenerative disorders by several years/decades. Up to 90.9% of patients with isolated RBD convert to DLB, PD or MSA. RBD might be part of the insidious neurodegenerative process, caused by the degeneration and Lewy body deposition instructures regulating REM atonia (223). The neurodegeneration follows the Braak staging: a gradual “rise” of pathological damage from brainstem areas to more rostral regions.

Patients with “isolated RBD” showing subtle abnormalities like hyposmia, important constipation, orthostatic hypotension, and/or neuroimaging features suggestive of neurodegeneration, are likely to have prodromal synucleinopathies.

RBD-related brain changes were detected with structural magnetic resonance imaging (MRI) and diffusion tensor imaging (DTI): microstructural changes in the white matter of the brainstem, the right substantia nigra, the olfactory region, the left temporal lobe, the fornix, the internal capsule, the corona radiata, and the right visual stream (224). The progression of RBD linked with parieto-occipital and orbitofrontal thinning as well as visuospatial loss, while the cognitive decline associated with parietal degeneration (225).

Isolated RBD patients' decreased striatal DAT binding (226), the loss of nigral hyperintensity on 3.0-T MRI and transcranial echo may predict short-term progress of RBD to synucleinopathy (227, 228).

Multimodal MRI, neuro-melanin-sensitive volume-, and signal intensity measures as well as fractional anisotropy discriminate RBD patients from controls and predict a Parkinsonian progress (229).

## RBD caused by autoimmunity: Anti-IgLON5 disease

Growing number of neurologic diseases have been recognized to have unexpected inflammatory or autoimmune etiologies e.g., PD and narcolepsy (230–232). A human leucocyte antigen (HLA) association is usually considered a hint to autoimmunity e.g., in narcolepsy, which is another REM sleep dysregulation syndrome sometimes overlapping with RBD and carrying the strongest HLA Class II association among all diseases. Also, RBD links with HLA class II genes: 84% of 25 RBD patients carried the DQw1 (DQB1^*^05,06) alleles and 28% were DR2 positive (233).

The recognition of a novel autoimmune-neurodegenerative disease-spectrum, the tauopathy anti-IgLON5 disease, indicates that an autoimmune etiology can cause RBD. Anti-IgLON5 disease is made by combinations of parasomnias, OSA syndrome, stridor, bulbar and limb movement disorders, axonal neuropathy and cognitive loss (234–241).

## RBD and PTSD

There is a peculiar combination of RBD with PTSD (242–245). A motor dysfunction with increased muscle twitches during REM sleep has been early noticed in relation to stress and PTSD, and a higher rate of RBD in PTSD patients compared to controls, even to trauma-survivors without PTSD, generating a distinct term - trauma-associated sleep disorder (TASD) (244), sharing the features of the two conditions.

In PTSD, the rate of stress-related norepinephrine turn-over of the LC might change, leading to norepinephrine depletion and even cell deaths shown by neuroimaging in humans. In rats, a single acute stressor could precipitate long-lasting changes in LC function. Due to the persistent decrease of LC noradrenergic output to the REM atonia network, RSWA and RBD may evolve (246, 247).

RBD in PTSD might be a good example of a psychological effect turning to organic (248–251).

## Antidepressants cause RSWA without RBD?

The association of RBD/RSWA with the use of selective serotonin reuptake inhibitors (SSRI) and selective norepinephrine reuptake inhibitors (SNRI) has been early described. Lately, several studies have shown an association of SSRI- and SNRI-use with RSWA only, curiously not accompanied by RBD (252–257). Patient manifesting episodes suspected for RBD, should discontinue all potentially related drugs, especially if taken at bedtime.

## RBD treatment

In line with the concepts described above, the approach to RBD treatment should initially focus on the removal of any reversible factor, as serotoninergic antidepressants, bupropion, alcohol and sleep-fragmenting conditions.

Even though most RBD treatment data were obtained by retrospective studies, case series, or small clinical trials with no high-level evidence, in clinical experience pharmacotherapy is widely used and it has changed little over the decade. Indeed, high dose melatonin (from 2 to 18 mg nightly) and clonazepam (from 0.5 to 1 mg nightly) may be effective, with the former better tolerated especially by the elderly (258).

## Sleep related painful erection

Sleep related painful erection is a rare parasomnia, occurring in 1% of men presenting with sexual problems.

It differs from the normal REM-related penile tumescence by the associated pain awakening the individual from sleep; who may have normal sexual life when awake. Local origins, a vagal dysfunction and central, especially antero-lateral hypothalamic etiologies have been raised. Baclofen has been found to be a good treatment option (259). Beta blockers, benzodiazepines and antidepressants were transitorily effective in some cases, and several additional treatments have also been used (260–262).

## Future directions

REM parasomnias make an informative group of sleep disorders; most of them are consistent with REM dissociation phenomena. Neurodegenerative and autoimmune/inflammatory conditions or stroke; brainstem compression syndromes as well as antidepressants or hypoxia may transitorily or permanently compromise the REM atonia network functioning. The different RBD syndromes may manifest specific symptom-patterns (distribution of the lack of muscle atonia) offering a future discrimination tool.

RBD, as an early sign of synucleinopathy, may carry a prognostic value providing the future opportunity of preventing it. NREM sleep features, especially CAP analysis, can help to improve the characterization of disease prognosis.

The special link of RBD with autoimmunity seems more than pure chance, given the important and shared HLA association of RBD and anti-IgLON5 disease, an autoimmune tauopathy. The overlap with narcolepsy, another REM sleep-related and autoimmune condition with HLA Class II association and REM sleep disturbance, needs further scrutiny.

The association of RBD with PTSD, forming together the new entity trauma-related sleep disorder, provides an example of a psychology impact turning to brain-organic.

Further scrutiny of REM parasomnias may provide data for understanding the role of antidepressants in sleep regulation; the link of depression (characterized with short REM latency) other REM disorders, and finally, help clarifying the functions of REM sleep.

## Circadian sleep-wake disorders

Humans are daily beings with inner rhythmicity bounded to the earth's day-night cycle. Circadian sleep-wake disorders (CRSWD) are caused by misalignment between internal sleep-wake rhythms and external synchronizers including, above all, the light-dark cycle. Circadian rhythm is physiologically supported by endogenously generated oscillations over a near-24-h period and continuously modulated by environmental cues and genetic predisposition (263). Individual rhythms are far from static conditions dynamically changing during the lifespan, due to hormonal changes and lifestyle habits, with a tendency for a first delay phase during adolescence followed by progressive shift into morningness in the adult age and among the elderly. The influence of parental control on sleep habits is assumed to be stronger during the early childhood, sometimes overshadowing the genetic predisposition (264).

## Chronotypes variability

Multiple internal and external factors influence individual chronotypes including genetic and epigenetic features, season of birth, photo-exposition during early perinatal period (265), ambient temperature, physical activity, food intake (266) and many others.

In the latest years population-based studies identified a progressive shortening of total sleep time and a circadian preference for eveningness in the adults and elderly, with even more evident differences among the youngest (267, 268), probably reflecting the impact of complex factors including the massive use of electronic devices, shifting in television programs schedule and job's related duties (268).

## The clinical spectrum of CRSWDs

The spectrum of CRSWDs include both “intrinsic” and “extrinsic” conditions. The firmer group is composed by advanced sleep-wake phase disorder (ASWPD), delayed sleep-wake phase disorder (DSWPD), non-24-h sleep-wake rhythm disorder (N24SWD), and irregular sleep-wake rhythm disorder (ISWRD). The so-called “*extrinsic” disorders (or environmentally related) involved the* shift work, jet lag disorder and other acquired “socially-related” irregularities of the circadian rhythm (269).

Light therapy may be used, according to individuals' phase response curves, to delay or to advance circadian timing. Typically evening light exposure, used before the core body temperature minimum (CBT-min), favors phase delays, whilst light therapy in the morning, after the CBT-min, leads to phase advances.

According to the task-force results, there is no evidence to support the use of strategic avoidance of light in patients with any CRSWD, while light exposure may be adopted only for patients with ASWPD and among patients with dementia and ISWRD (“weak for” recommendation).

Sleep promoting medications should always be avoided in patients with CRSWD, especially among patients suffering for dementia (“strong against” recommendation).

Timed oral melatonin administration could be adopted in patients with DSWPD with or without psychiatric comorbidities; N24SWD or children/adolescent with ISWRD (“weak for” recommendation), however the same strategy should be avoided in elderly with dementia and ISWRD (“weak against” recommendation).

Wakefulness promoting medications and/or somatic interventions should be avoided and combined therapy (light therapy + multicomponent behavioral interventions) could be used for children/adolescents and DSWRD (“weak for” recommendation).

Notably, the summarized practice guidelines rely on few and heterogeneous published researches on CSWRD and, so far, the scenario still appears blurred due to insufficient/absent data.

Knowledges pitfalls in CSWRD had nicely been analyzed in an international workshop (270). Among the major gaps in the field there is the absolute lack of individualized evaluation. Even if few sleep centers use the salivary dim light melatonin onset (DLMO) as indicator of circadian phase, currently no standardized protocol exist and CSWRD diagnosis is based on self-reported sleep habits and schedule. No exam or biological biomarker is required to measure circadian timing in patients with suspected CRSWD, to tailor disease strategy on patient's characteristics.

Further researches are urgently needed to investigate diseases phenotypes and their evolution with aging.

## Smart working and sleep disturbances

In the latest months, as direct social consequence of the COVID-19 pandemia, a specific kind of work schedule organization impressively increased: the smart working, whose long-lasting impact on wake-sleep rhythm is to be defined. Intuitively patients suffering from pre-existent intrinsic circadian sleep disorders could have gain few benefits from this new situation, due to higher flexibility on their schedules, bedtimes schedule and spare time organization. However, the reduct exposition to external synchronizers (light, activities, sports) and the increase exposure to artificial light and electronic devices, frequently until late in the night, may have favored misalignment of individuals circadian rhythm. Data collected from the International Labor Organization (ILO) pointed out some other negative effects of smart working, including a tendency to work longer and an overlap between paid work and personal life, which can lead to high levels of stress (271). We assume that some gender-related differences may exist, being women, especially if with kids, more prone to overwhelming duties and with a tendency for multi-tasking activities.

To what extent the forced novel scenario of “smart-work” have influenced people with intrinsic CSWRDs and/or whether it has promoted novel categories of extrinsic circadian sleep-wake diseases need to be investigated.

## Shift working

Another category of workers overexposed to circadian irregularity/misalignment are those practicing shift work, a condition associated with a recurring work schedule that overlaps the usual time for sleep (24). The full spectrum of shift work comprises regular evening or night schedules, early morning shift work, evening/afternoon shift work, rotating shifts, split shifts, on-call or casual shifts, 24-h shifts, irregular schedules, and other non-day schedules. Impact on sleep quality and amount could be different according to shift types, with morning shifts frequently associated to REM sleep loss and severe sleep inertia (272); night shifts usually associated with greater curtailment of total sleep time and rotating shift schedules facing additional challenges related to the speed and direction of shift rotations (273). Shift workers are at higher risk for car crashes (274), behavioral changes (275), metabolic diseases (276, 277), cardiovascular diseases (278, 279), hypertension (280, 281), infertility (282) and tumors. Abnormalities in clock genes expression had been associated to miscarriage and infertility in women, impacting hypothalamic regulation (283), with still inconclusive data regarding fertility impact in male.

Genetic differences are assumed to be involved in terms of vulnerability toward sleep deprivation (284). Resistant subjects tend to respond more efficiently to unanticipated sleep–wake states (285). Factors associated to shift work tolerance include young age, eveningness, extraversion and male sex (286).

## The circadian rhyhtm scenario in the youngest and the oldest

The younger face also another form of external circadian stress, which appears strongly related to social demands. As a consequence many subjects find themselves living a novel form of jet-lag: the “social jet-lag”, due to continuous changing in sleep-wake habits from week and weekend, frequently leading to coping strategies such as soft drinks, caffeine and alcohol consumption or abuse (287). On the opposite side the modern society needs to tackle with the progressive demographic aging of the population, ending in the continuous increase of the number of subjects with major frailty toward circadian rhythms disruption, partly due to the aging-dependent functional decline in light sensitivity and sedentary lifestyle (288). Sleep among older individuals is characterized by reduced percentage of SWS, increase of light sleep stages and multiple nocturnal awakenings (289). Animals studies suggests that several clock genes expression is reduced with aging (290). The scenario is even more severe among patients with neurodegenerative disorders such as Alzheimer's disease (AD), where circadian misalignment is more extreme due to mechanisms related to the neurodegeneration process itself (loss of the circadian “pace-maker” vasoactive intestinal peptide-expressing neurons in the CNS, direct effects of amyloid on clock genes expressions and other hypothesized factors) (291), to virtually absent exposition to external synchronizers and to manifold medication side effects (292). Circadian sleep disruption can be revealed since the earlier prodromal phase of Alzheimer's disease, preceding cognitive manifestations and, in untreated, may faster the progression of neurodegeneration (293). Same derailment in sleep-wake regularity concurrently involved AD patients' caregivers, whose schedules are driven by patients' needs (294) and whose caring capability could be strongly hampered by circadian misalignment.

## Health consequences of circadian rhyhtm disruption

Sleep-wake disruption may lead to increase risk for adverse mental health outcomes, with stronger impact on emotional control among women, exposed to higher risk for depressive symptoms (295) and higher risk for maladaptative behaviors, including suicidal ideation, among men (296). The relationship between circadian rhythm and mental health is complex and bidirectional, as supported by consolidated evidences confirming the role of chronotherapy as powerful treatment for unipolar and bipolar depression (297). Besides the known consequences of circadian rhythm irregularity on mood stability and cognitive functions, there are also some others regarding metabolic balance (298). Adolescent with social jet-lag are at higher risk for obesity, with female more exposed with respect to young male (299). Circadian misalignment significantly decreases the levels of circulating anorexiant hormone leptin, with an in-parallel increase of the appetite-stimulating ghrelin (300). Clocks genes are involved into cell proliferation and oncoregulation (301) and melatonin itself act as antioxydative and onco-suppressant agent and converging evidences highlighted the existence of stronger risk for certain types of tumors (breast, thyroid, lung, prostate, pancreas tumors and others) (302) in subjects experiencing circadian disruption (303). Factors involve including melatonin and estrogen fluctuations, chronic inflammation, autoimmune dysfunction, metabolic impairment and DNA oxydative damage (302). Chronodysruption is recognized as an independent pro-oncogenic risk factor (Level of evidence IARC (International Agency for Research on Cancer) (2A), with risks increase with duration of exposition to work-shifting (302), being higher after 20 years.

## Future directions

In summary circadian imbalance needs to be handled in a comprehensive view, as many factors can be involved in its modulation/dynamics (304). A more individualized approach for intrinsic CSWRDs is desirable. Biomarkers for a better definition of circadian sleep-wake cycles are advisable. There is still lack of knowledge on the relationship between CAP and CSWRDs. It might be interesting to verify the utility of CAP metrics in the follow-up of CSWRDs diagnosis and follow-up.

Finally, in a more “olistic” perspective, it is probably more correct to refer to a “circadian syndrome” rather than “circadian sleep disorder”. Long-term prospective data on health consequences of smart working are advisable.

## Brain imaging and sleep disorders

### Advance in brain imaging

Brain imaging technologies gained impressive progress in the latest years, permitting some attractive breakthroughs in both structural and functional central nervous system analysis.

The availability of advanced computer-aided tolls to support qualitative evaluation including voxel and surfaced-based morphometry, DTI white matter reconstruction, shape analysis and others, lead to outstanding growth of knowledges about brain tissue characteristics, dynamics and functioning. Modern post-processing methods permit to explore brain anatomy beyond the resolution of naked-eye image observation, allowing to analyze MRI frames with ultra fine resolution, exploring neural connectivity profile and structural-functional coupling (305, 306).

The apparent paradox of using “structural” data to analyze connectivity profiles had already been tackled in multiple research fields, including above all epilepsy and psychiatry (307, 308). Structural networks could be inferred from diffusion MRI tractography or inter-regional covariance patterns of structural measures such as cortical thickness or gyration, extending the significance of morphometric studies to functional rationale. In these frameworks the identification of high morphological correlation between distinct regions (“covariance”) can be interpreted as indirect sign of networks union (309). Besides automated and semi-automated advanced structural MRI processing, variable functional methods including static and dynamic fMRI (functional-MRI), task-related or task-free investigations are also widely used.

## Brain imaging in sleep disorders

So far, the utilization of advanced structural or functional brain imaging for sleep disorders is still limited to research purposes, with no application in everyday practice. Indeed, most of described post-processing techniques are strongly demanding and time-consuming, requiring dedicated training formation. Besides sleep disorders diagnosis rarely even require the execution of brain imaging of any sorts, with some minor exceptions including among others sleep-related epilepsies, RBD or narcolepsy. And, even in these cases, brain MRI results are not taken into account in the disease's diagnostic criteria, being useful instead to look for or to rule out the existence of underlying structural lesions and current sleep disorders classification is based only on clinical and/or polysomnographic criteria. We argued if imaging informations could support a more tailored approach toward sleep pathology and sub-classification.

Nowadays one of the major drawbacks of imaging studies in the sleep disorders field is the lack of standardized imaging protocol for acquisition or processing of brain images. Important differences in studies' results may thus reflect methodologies and acquisition differences, hampering results' comparisons and depleting conclusive considerations (310). Moreover, the absence of normative data from healthy sleepers and the small samples of most of available studies affect the interpretation of results.

The creation of open-source big dataset/atlas from healthy sleepers with univocal imaging protocol for acquisition is recommended and could support the continuous increase of expertise in this fascinating field (311).

The sleeping brain organization dynamically changes at variable levels across lifespan, as demonstrated both with PSG recording and at clinical level. At the same time brain structure itself dynamically evolves with aging, following non-stochastic trajectories and respecting hierarchical dynamics (312, 313) according to neurodevelopmental growth. Given the relevance of sleep for brain functioning, memory performance and learning, the measurement of its impact on anatomic and functional brain dynamics is not a trivial issue.

Some common sleep disorders could act as facilitators for premature brain aging (e.g., insomnia, or sleep breathing disorders), leading to potentially severe cognitive consequences (314, 315). This is not surprising due to the demonstrated role of sleep in terms of body and mind restoration, toxins elimination (such as Ab and tau) (316, 317) synaptic strengthening and pruning, endocrine balance and autonomic correct functioning.

Sleep duration influences the amount of cortical deposition of Ab in healthy sleepers (318). Thalamus and hippocampus concentration of Ab increases after only one night of acute sleep deprivation (319) and cerebrospinal fluid levels of Ab42 are significantly increased in chronically insomniac patients (319). Morphometric studies showed that chronic insomniac patients present lower brain volumes in several cortical and subcortical brain areas including medial frontal and middle temporal gyrus, middle cingulate cortex, basal ganglia and hippocampus as well as gray matter increase in the anterior cingulate cortex (320), with changes correlated with disease severity. Insomnia may thus exacerbate the neuropathological processes involved into cognitive impairment (321). Given the wide heterogeneity of insomnia subtypes we argued if the utilization of imaging informations could provide some novel insights into disease mechanism, helping in stratifying patients' severity according to the extent of brain damage. Unfortunately, the lack of standardized MRI protocols and the small sample sizes of most studies exploring insomnia morphometric data account for several inconsistencies across studies' results (322).

## Imaging insight into sleep disorders

RLS and PLMD are the commonest sleep-related movement disorders, frequently overlapping in the same patient. Whilst RLS pathogenesis is assumed to derive from a dysfunction of the dopaminergic hypothalamo-spinal inhibitory pathways and a frequent relationship with reduced brain iron content (323) and spinal hyperexcitability is a consolidated issue (324), pathogenesis of PLMD is still largely unknown. Imaging studies in RLS patients revealed the presence of decreased iron content in the substantia nigra, but not in other subcortical nuclei (caudate head, putamen, globus pallidus, red nucleus) among late-onset RLS (325). Conflicting results were found in similar studies covering the topic (326, 327), probably once again reflecting inconsistencies in patients' selection (on medication/drug free; age of participants; disease duration), MRI scan strength (1.5 Tesla vs. higher field machines) or methodologies (manual tracing ROI, automated ROI identification and others).

Neuroimaging investigations exploring isolated PLMD neurobiology are lacking. Higher score on PLM index has been associated with increased risk for white matter hyperintensity burden and thus has been proposed as a neuroradiological biomarker for increased risk of cerebrovascular events (67, 328). Data from HRV analysis revealed the association of PLMD with reduced parasympathetic tone, increased sympathetic tone (49), supporting its role as an acquired (and manageable?) risk for cardiovascular events. Secondary forms of PLMD has been described in association with pontine stroke (329, 330), supratentorial stroke (331) and parietal hemorrage (332), leading to the hypothesis that the loss of cortical or subcortical inhibition on brainstem movement generators (e.g., reticular formation) might cause PLMD. Further imaging studies are advisable to enhance our knowledge on both PLMD pathogenesis and its impact on cardiovascular risk.

Furthermore, given the lifespan dynamics of PLM index itself, dedicated investigations should be performed to ease the discrimination of more “disturbing” phenotypes requiring treatment, from those reflecting the para-physiological aging of neuro-spinal networks.

Brain imaging is currently not recommended for SHE diagnosis. However, numerous SHE patients, according to the International League Against Epilepsy (ILAE) diagnostic criteria (333), can be described as affected by focal epilepsy with a structural etiology, as malformations of cortical development (MCDs) and low-grade associated tumors (LEATs) are among the commonest radiological findings. Even if traditionally associated with frontal-lobe onset, due to its strong association with abrupt hyperkinetic automatisms and complex behaviors, it has been widely confirmed that the seizure onset zone in SHE patients may also be extra-frontal (334). The subsequent spreading of epileptiform discharges to the anterior cingulate and frontal areas, irrespectively of its origin, explain the well-known semiological similarities between patients with distinct seizure onset zone (SOZs). Reduced N-Acetyl-aspartate/Creatine (NAA-Cho) ratio had been identified in the anterior-cingulate cortex of SHE patient using proton magnetic resonance spectroscopy (335). Notably the same NAA-Cho reduced ratio had been associated with neuronal loss, abnormal metabolism and gliosis in temporal lobe epilepsy patients (336). At functional level SHE patients present higher functional connectivity between thalamus and sensori-motor cortices, areas directly involved in the K-complex dynamics and arousal pathway. The intrusive role of arousal in the SHE pathogenesis is a well-known issue. Brain imaging results may support a deeper understanding of mechanisms linking sleep-instability, arousals and SHE-related epileptiform networks in a comprehensive framework.

Another challenging scenario for the sleep clinician is the one of hypersomnolence disorders e.g., narcolepsy type 2 or idiopathic hypersomnia (IH); both conditions' diagnosis being often tricky. Even if underlying medical conditions should always be ruled out, no formal recommendations impose brain imaging as part of the diagnostic work up.

New-onset hypersomnia may be the first clinical hint for inflammatory brain lesion (337), vascular events (338) and numerous other conditions. Previous works highlighted the presence of subtle structural abnormalities at MRI visual inspection among around 1/3 of patients suffering from idiopathic hypersomnia and studied with conventional brain MRI, but with significant clinical impact on patient management just in a minority of cases (339).

In the latest years growing attentions has been dedicated to the “task-free” connections within the brain. Accordingly spontaneous low frequency neural oscillations may be traced to recognize functionally linked (but anatomically separated) brain areas, referred as resting state networks (RSN) (340).

RSN-f-MRI studies identified further consequences at both structural and functional levels in most patients with IH including “thicker” cortex in pivotal nodes of one of the most important RSN, the default mode network (DMN), at the level of precuneus and medial prefrontal cortex, together with decreased functional activation of its anterior part (341). A strong covariance tendency/pattern within the entire DMN had also been described in patients with IH. Similar results were confirmed in functional SPECT studies (342), suggesting a network-wide level for the pathology. EDS has also been associated with regional A-beta amyloid deposition in the anterior cingulate, posterior cingulate-precuneus and parietal regions in an elderly cohort with no cognitive deficits (343), confirming the involvement of DMN nodes in hypersomnolence appearance. In acutely sleep deprived subjects sleepiness was associated with distinct anterior-to-posterior gradient of hypo-activation of the DMN (344) and in Narcolepsy type I sleepiness severity was linked to reduced functional connectivity measures in the left anterior cingulate gyrus and in the bilateral posterior central gyrus (345). The antero-medial part of the DMN is strongly connected with the ascending activating system and represents one the most important networks for alertness and self-consciousness (346). Aberrant connections between DMN and other resting state netwroks such as the salience network, has been described among insomniac patients (347). Hopefully advanced investigations on DMN subdivisions and connections could provide a deeper understanding of alertness and sleepiness mechanisms and of sleep pathologies.

Brain organization dynamics during NREM sleep stages had also been recently explored (348), with some interesting novelties regarding networks' interactions evolution with respect to sleep depth. According to this study interdependencies and interactions between networks progressively increase during the “unstable” stage N2 and subsequently break down with the appearance of the more “stable” stage N3.

Multimodal EEG-fMRI investigations may also help in answering to some fascinating open-questions such as reasons explaining inter-individual differences in dream recall frequency and capacity (349). Enhanced connectivity profile within the DMN and regions involved in memory processing seems to be important to promote short term memory recall during sleep-wake transitions.

In this direction advanced neuroimaging studies could empower our understanding of basic neurophysiological mechanisms embedded into sleeping brain functioning.

## The forgotten sleeping cerebellum

Besides cortical and subcortical regions, even more mysterious is the involvement of other relevant brain structures in sleep texture organization and sleep disorders, such as, above all, the cerebellum. Regardless to the central role of sleep in consolidating procedural learning and the major significance of the cerebellum in the development of motor performances surprisingly, so far, only few efforts have been dedicated to understand the dynamics of cerebellum during sleep. Changing the firing patterns during NREM and REM sleep in cerebellum cells is well-documented (350, 351). The cerebellum also express various genes involved in the circadian rhythm organization (352). Sleep spindles are associated to BOLD signal in the cerebellum in fMRI studies (353) and bidirectional influences in neural firing of thalamo-cortical output and cerebellar cortex have been documented in animals (354). Variations in cerebellum morphometry and volume have been identified in numerous sleep disorders including insomnia, OSA or hypersomnolence disorders (355). Cerebellum appears implicated also in RBD pathogenesis with controversial morphometric results (356, 357).

Given the technical difficulties in examining cerebellum EEG activity with surface recording (the near-by occipital visual areas lead to several recording artifacts), probably more reliable informations could be collect through advanced brain imaging analysis (358). Further studies are advisable to allow for a deeper understanding of the complex relationship between sleep, sleep disorders and the cerebellum.

## Future directions

The proper characterization of sleep diseases, referring only to disease's clinical features may be challenging and, sometimes, incomplete. The creation of big dataset from healthy sleepers of variable ages and the definition of shared images protocol for acquisition are mandatory to guarantee progress in this fascinating field of research.

Information collected from brain imaging may provide support for disease's diagnosis, enhance understanding regarding disease's neurobiology and lead to extremely sensitive follow-up with respect to disease's evolution and consequences.

Dedicated education for advanced brain imaging management should therefore be proposed to sleep clinicians and, equally important, adequate training regarding sleep disorders and their impact in brain morphometry and imaging should involve neuro-radiologists.

## Sleep recording methodology

The overwhelming richness of biological information encoded in sleep state data is largely minimized by conventional approaches of assessment. This “simplification”, though having some utility in the realms of pragmatism and convenience, has severely impaired the cognitive landscape of thinking and using sleep data. Advances in visual and computational analyses now offer opportunities to improve disease characterization and endotype/phenotype characterization, applicable to a range of sleep disorders.

## Methods of characterizing sleep

The scoring manual of the AASM accessible through the Internet (The AASM Manual for the Scoring of Sleep and Associated Events. http://www.aasmnet.org/scoringmanual/default.aspx) provides standard but simplistic scoring guidelines generating sleep stage, heart rate, breathing, and motor activation metrics. However, besides the standard stages, alternate methods of characterizing sleep include mature technologies such as high resolution spectral analyses, a continuous sleep depth measure called the Odds Ratio Product (ORP) (359, 360) and variants thereof, CAP of NREM sleep (361) and CPC (high, low and very low frequency coupling of autonomic and respiratory drives, modulated by cortical delta power) (362). NREM stage N3 is usually associated with stable breathing. However, such periods frequently occur outside N3, during a stable form of N2, when the EEG usually shows a non-CAP morphology. Arousals are traditionally scored as an all or none event but show a wide range of perturbation and recovery kinetics, and individual differences which may amplify or dampen the progression of sleep state or respiratory events (363–367). A number of computational methods are available to identify high loop gain of the respiratory control system, which is of key therapeutic importance.

## High quality EEG spectral analysis

Spectral analysis of the sleep EEG has a long history of use in sleep research but not in clinical practice. Part of the reason is the “fuzziness” of the graphs generated by conventional polysomnographic software, making reading/interpretation difficult. Multitaper is a spectrum estimation method developed to provide an optimal tradeoff between time and frequency resolutions using taper functions, which are called Slepian sequences. Each taper provides a different emphasis of the signal. Each tapered signal undergoes Fourier transform, which converts signal (time domain) into a spectrum (frequency domain). The multitaper spectrum estimation means to average these spectra, which leads to an unbiased and less noisy spectrum. The strength of the multitaper spectrum estimation is due to the fact that the taper functions are orthogonal to all others. Therefore, each tapered signal is focused on specific spectral aspects, and after averaging it combines these different aspects while not canceling each other (368).

## CPC maps sleep stability domains

The SleepImage^®^ System (369) performs CPC analysis and can be run on any signal recordings that include an ECG or similar information-content signal including pulse plethysmography, including full or limited polysomnography. The system enables ambulatory assessment of sleep quality, actigraphy, heart rate measures, and sleep apnea (23). The current embodiment of the technology uses Ring Oximeter coupled to a Smartphone Application linked to a Cloud Computing system. There are no disposable elements, and the Ring enables easy repeated testing. The Ring can record for 16 h on a single charge. The raw pulse photoplethysmogram can also be analyzed for arousal events.

CPC analysis may be performed on any signal which has heart rate and respiratory tidal volume information, including the electrocardiogram and the pulse photoplethysmogram (PPG) (370, 371). Two distinct patterns of CPC are observed: high frequency coupling (HFC; 0.1–0.4 Hz) and low frequency coupling (LFC; 0.01 to < 0.1 Hz). HFC is associated with stable breathing during NREM sleep. HFC aligns with critical biological structures of sleep, including delta power, stable breathing, blood pressure dipping, and an unfragmented < 1 Hz slow oscillation (23, 372). A preponderance of a subset of LFC, named elevated-low frequency coupling (e-LFC), detects apneas and hypopneas (373).

By detection and quantifying self-similar sinusoidal oscillations of HRV typically seen in periodic breathing/Cheyne-Stokes narrow spectral band e-LFC (e-LFC_NB_, putative central sleep apnea, periodic breathing, or complex sleep apnea) is determined (374). A third CPC pattern named very low frequency band (v-LCF; 0 to < 0.01 Hz) has two subsets, wake and REM sleep. When classifying REM vs. wake, spectral dominance in the upper range of the v-LFC band in addition to lack of motion or motion artifact, is indicative of REM sleep. Total sleep time (TST) and sleep efficiency are then determined as follows: (i) Establish actigraphic sleep boundaries. (ii) mark the sleep onset as the first occurrence of HFC or LFC, (iii) mark the sleep conclusion as the last occurrence of HFC or LFC, or the last occurrence of REM, and (iv) classify the windows of v-LFC dominance into either REM or wake.

Assessment of CPC parameters and the biological correlates have been performed in numerous conditions, including sleep apnea pre/post treatment (23, 375), insomnia (376), depression (377), fibromyalgia (378), stroke (379), atrial fibrillation recurrence risk (380), glucose handling (381), and blood pressure regulation (382). The technology is valid in children as well (383, 384). A FDA-approved polysomnogram-equivalent apnea-hypopnea index is now available (368).

## Sleep state modifies the expression of sleep apnea

Airflow patterns and arousal thresholds are modulated by sleep macrostructure (REM and NREM sleep, stages of NREM sleep), sleep microstructure (CAP and non-CAP), homeostatic sleep drive, medication, and age. Data suggest that NREM sleep is bimodal rather than graded, with stable and unstable regime, or alternatively conceptualized as effective and ineffective. Thus, rather than thinking in terms of grades of NREM sleep (N1–N3) of progressive depth, the stability domain has only 2 forms of NREM sleep—stable and unstable. N3 is usually stable, N1 is always unstable, but N2 may be stable or unstable. These periods of stability intrinsic to NREM sleep can determine the instantaneous presence or absence of sleep apnea. Cardiopulmonary analysis shows that stable breathing periods result in high frequency coupling of respiration and HRV, simultaneously associated with high EEG delta power. Such periods are not restricted to N3 but make up most of N2 in health. Intermittent periods of stable breathing are well-recognized in patients with even severe obstructive sleep apnea, during both N3 and N2 (385). Flow-limitation can be prominent, and both hypoxia and hypoventilation may occur during these obstructed but stable periods with prolongation of inspiratory time with minimal breath-to-breath variability of tidal volume. These periods do have an impact on the EEG, but visual determination is not possible, and computational methods such as the respiratory cycle-related EEG change are required (386, 387). The persistence of CAP-type features on the EEG may also occur during SWS associated with severe persistent flow-limitation, suggesting the presence of concomitant disruptive influences from hypoxia, hypercapnia, excessive respiratory effort, and upper airway stimulation.

Excessively unstable NREM sleep disproportionate to disruptive pathology on the PSG may be recognized by a sleep fragmentation phenotype. This phenotype can be suggested by prolonged sleep-wake transitional instability (>10 min), low sleep efficiency (< 70%), persistently high N1 stage during positive airway pressure (PAP) titration (>15%), and poor evolution of SWS (< 1 Hz) (363). In contrast, even severe sleep apnea may coexist with relatively well-preserved architecture. Chin EMG tone elevations during NREM sleep are not part of the conventional arousal definition, but the duration and degree of elevation vary markedly between individuals and stays consistent within individuals. The same concept can be applied to the EEG (arousal duration or return to sleep) and heart rate responses to arousal—inter-individual variability is contrasted with intra-individual stability (388).

## Sleep is a continuous flow, which can be perturbed and distorted

The continuity of sleep during individual sleep cycles across a typical night requires networking, cohesion, and reciprocal interactions of NREM and REM sleep processes. Intuitively, stable periods of NREM sleep are deeper, but both stable and unstable NREM sleep can have varying degrees of depth. Conventional stages do not effectively capture sleep depth, even if in most instances N3 is deeper and more stable than N2.

One established method of assessing continuous sleep depth is the Odds Ratio Product (389). ORP is calculated every 3 s from the power spectrum of the EEG. It represents the EEG powers in four different frequency bands relative to each other, with higher values representing increased propensity for an arousal. It has an excellent correlation (*r*^2^ = 0.98) with arousability, defined as the probability of an arousal or awakening occurring within 30 s. It also changes appropriately in response to sleep deprivation, sleep restriction, and brief acoustic stimuli, and increases across the night, as may be expected from the decrease in homeostatic sleep pressure. A related method using machine learning approaches provides a wider and intuitive range of numerical values, the Ordinal Sleep Depth, with profiles very similar to the ORP.

Sleep depth post-arousal varies substantially among subjects and patients with OSA from being very light to very deep. The ORP fluctuates after an arousal, rising to wake levels and then decreasing as sleep resumes and stabilizes. ORP-9 is the ORP 9 s after the start of the arousal (390). If the ORP remains high several seconds after the arousal, it suggests that the “glue” (network cohesion) of sleep is poor, and this high ORP-9 would indicate that the high arousability is much more likely to be due to abnormal central regulation of sleep depth. In patients with high ORP-9 (highly arousable soon after a previous arousal), arousal is likely to occur soon after the obstruction and interrupt the recruitment of pharyngeal dilators, leading to recurrence of events. Conversely, subjects in whom sleep becomes quite deep immediately after arousal (low ORP-9) can tolerate the obstruction for a longer time allowing the reflex recruitment of dilator muscles, thereby increasing the probability of resolving the obstruction without arousal. ORP-9 was found to be the main determinant of average sleep depth and to be invariably high in patients with very high AHI. Furthermore, ORP-9 is quite reproducible even when re-measured after 5 years, suggesting that it is a trait (Younes M; unpublished observations).

## A vertically integrated approach to sleep data analysis

**Figure 6:**
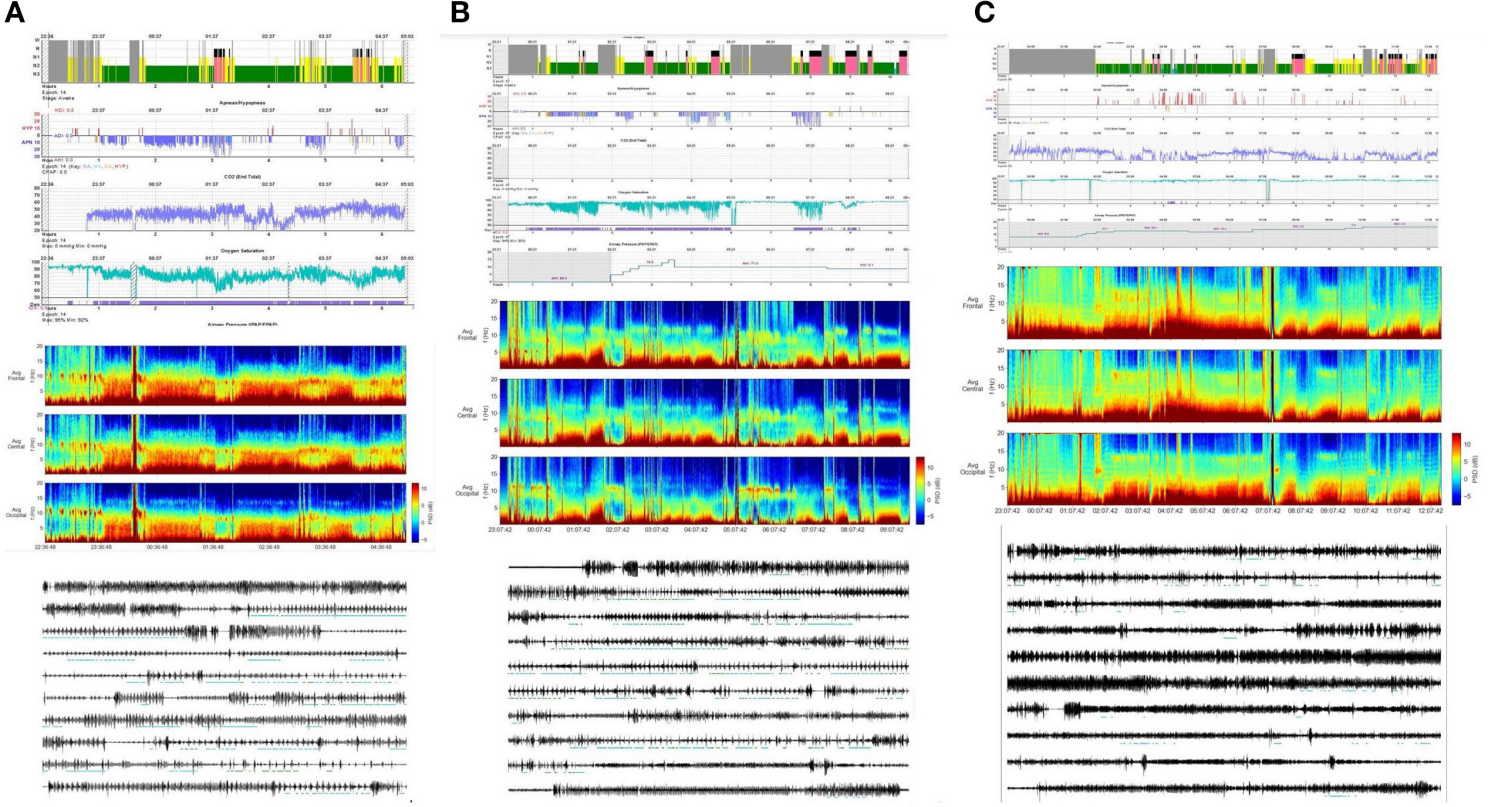
Three examples showing that value of vertical integration of high-quality EEG spectral analysis inclusive of respiration, oximetry, capnometry, and respiratory phenotype in OSA patients. **(A)** Patient with intrinsically high sleep quality, who develops severe sleep apnea and high loop gain, complicated by hypoventilation. Merely supporting the upper airway with continuous positive airway pressure will improve oxygenation and ventilation, but respiratory instability is likely to persist. Aggressive ventilation could amplify periodic breathing. In this case, a contribution from sleep fragmentation will be minor, given the high delta power despite the highly disruptive pathology. **(B)** Delta power profile is highly disrupted, and initial sleep apnea treatment is not effective. Once high loop gain is targeted (oxygen and dead space), sleep and sleep apnea markedly improves. **(C)** Sodium oxybate is administered (3 gm) just before lights out, but the patient remains awake for over 2 h. Once he falls asleep, the large increase in slow-wave power typical of oxybate sleep effects in not seen, but rather, sleep stage and sleep powers remain fragmented. Slow-wave power increases late, suggesting that there is substantial circadian phase delay. The drug does cause an increase in delta power while awake, before sleep onset. This fragmentation is contributed to by difficult to treat sleep apnea, and sleep fragility of uncertain cause. This patient has a diagnosis of hypersomnia in addition, being able to sleep 16+ h in a 24-h period. Even after the second dose of oxybate (3 grams), sleep remains fragmented. Stable breathing is relatively low, but there is no pervasive periodic breathing / high loop gain apnea as in the other two examples.

Every method assessing sleep, provides unique information on sleep architecture in health and disease. How does one reconcile these various methods? A new approach to sleep data is proposed. Rather than various methods being competitive or additive at best, it is proposed that by “stacking” and “vertical integration”, profound new insights may be obtained. The examples below show the strength of this “insight at a glance” approach (Figure 6).

## Author contributions

LP, CM, PH, AS, and RJT conceived of the presented idea and drafted the manuscript. NA, FR, SP, AZ, and FM contributed to the final version of the manuscript. All authors have seen the manuscript and approved it in its current version.

## Conflict of interest

The authors declare that the research was conducted in the absence of any commercial or financial relationships that could be construed as a potential conflict of interest.

## Publisher's note

All claims expressed in this article are solely those of the authors and do not necessarily represent those of their affiliated organizations, or those of the publisher, the editors and the reviewers. Any product that may be evaluated in this article, or claim that may be made by its manufacturer, is not guaranteed or endorsed by the publisher.

## Glossary

(N)REM sleep, (non) rapid eye movements sleep; AASM, American Academy of Sleep Medicine; Ab, beta-amyloid; AD, Alzheimer's disease; AHI, apnea–hypopnea index; ALS, amyotrophic lateral sclerosis; ASWPD, sleep-wake phase disorder; BMI, Body max Index; BOLD, blood-oxygen-level-dependent; CAP, Cyclic Alternating Pattern; CBT, Cognitive behavioral therapy for insomnia; CBT-min, core body temperature minimum; CNS, central nervous system; COMISA, Comorbid Insomnia and Sleep Apnea; COPD, chronic obstructive pulmonary disease; CoSA, Comorbidities of Sleep Apnea; CPC, cardiopulmonary coupling; CRSWD, Circadian sleep-wake disorders; CSF, cerebro-spinal fluid; DLB, diffuse Lewy body disease; DLMO, light melatonin onset; DMN, default mode network; DOA, Disorders of arousal; DSWPD, delayed sleep-wake phase disorder; DTI, diffusion tensor imaging; EDS, excessive daytime sleepiness; EEG, electroencephalogram; ESADA, Sleep Apnea Network/European Sleep Apnea Database; fMRI, functional MRI; GABA gamma-aminobutyric acid; GP, general practitionerice; HD, Huntington's disease; HLA, human leucocyte antigen; HRV, Heart rate variability; IARC, The International Agency for Research on Cancer; IEDs, interictal epileptiform discharges; IFL, inspiratory flow limitation; IH, idiopathic hypersomnia; ILAE, International League Against Epilepsy; ILO, International Labor Organization; IMI, intermovement interval; ISI, insomnia severity index; ISWRD, irregular sleep-wake rhythm disorder; LMs, Leg Movements; MCDs, malformations of cortical development; MRI, magnetic resonance imaging; MSA, multisystemic atrophy; N24SWD, non-24-hour sleep-wake rhythm disorder; nAchR, nicotinic acetylcholine receptor; ORP, Odds Ratio Product; OSA, obstructive sleep apnea; PAP, Positive Airway Pressure; PC, pre-coeruleus region; Pcrit, Pharyngeal critical closing pressure; PD, Parkinson's disease; PER2, Period Circadian Regulator 2; PI, Periodicity Index; PLMD, periodic leg movement disorder; PPG, pulse photoplethysmogram; PSP, progressive supranuclear palsy; PTSD, post-traumatic stress disorder; PWA, pulse wave amplitude; RBD, REM sleep Behavior Disorder; REM_rapid eye movement; RLS, restless leg syndrome; RSD, restless sleep disorder; RSN, resting state networks; RSWA, REM sleep without atonia; SHE, Sleep related hypermotor epilepsy; SLD, sublatero-dorsal nucleus; SNRI, selective norepinephrine reuptake inhibitors; SSRI, selective serotonin reuptake inhibitors; SWS, slow wave sleep; TASD, trauma-associated sleep disorder; TDP, TAR DNA binding protein; UARS, upper airway resistance syndrome; WASM, Word Association of Sleep Medicine.
